# Proteomic Analysis Reveals Key Proteins and Phosphoproteins upon Seed Germination of Wheat (*Triticum aestivum* L.)

**DOI:** 10.3389/fpls.2015.01017

**Published:** 2015-11-18

**Authors:** Kun Dong, Shoumin Zhen, Zhiwei Cheng, Hui Cao, Pei Ge, Yueming Yan

**Affiliations:** ^1^Lab of Molecular Genetics and Proteomics, College of Life Science, Capital Normal UniversityBeijing, China; ^2^Hubei Collaborative Innovation Center for Grain IndustryJingzhou, China

**Keywords:** bread wheat, seed germination, 2D-DIGE, proteome, phosphoproteins

## Abstract

Wheat (*Triticum aestivum* L.) is one of the oldest cultivated crops and the second most important food crop in the world. Seed germination is the key developmental process in plant growth and development, and poor germination directly affects plant growth and subsequent grain yield. In this study, we performed the first dynamic proteome analysis of wheat seed germination using a two-dimensional differential gel electrophoresis (2D-DIGE)-based proteomic approach. A total of 166 differentially expressed protein (DEP) spots representing 73 unique proteins were identified, which are mainly involved in storage, stress/defense/detoxification, carbohydrate metabolism, photosynthesis, cell metabolism, and transcription/translation/transposition. The identified DEPs and their dynamic expression profiles generally correspond to three distinct seed germination phases after imbibition: storage degradation, physiological processes/morphogenesis, and photosynthesis. Some key DEPs involved in storage substance degradation and plant defense mechanisms, such as globulin 3, sucrose synthase type I, serpin, beta-amylase, and plastid ADP-glucose pyrophosphorylase (AGPase) small subunit, were found to be phosphorylated during seed germination. Particularly, the phosphorylation site Ser^355^ was found to be located in the enzyme active region of beta-amylase, which promotes substrate binding. Phosphorylated modification of several proteins could promote storage substance degradation and environmental stress defense during seed germination. The central metabolic pathways involved in wheat seed germination are proposed herein, providing new insights into the molecular mechanisms of cereal seed germination.

## Introduction

Wheat (*Triticum aestivum* L.), the second most important food crop in the world, has high nutritional value and is rich in starch, protein, fat, minerals, calcium, iron, thiamin, riboflavin, niacin, and vitamin A (Šramková et al., 2009). Wheat seeds consist mainly of starch (up to 70%) and proteins (12–15%). Starch is generated from carbohydrates, which is activated to provide energy during seed germination. The proteins in wheat seeds can be divided into albumins, globulins, gliadins, and glutenins. Albumins and globulins are important enzymes that take part in plant growth (Wiesner et al., 1980). Gliadins and glutenins are the major storage proteins in wheat endosperm, which confer extensibility and viscoelasticity of dough and affect the processing quality of wheat (Payne, 1987).

Seed germination, a critical process for plant propagation, starts by seed imbibition, causing the embryo to transition from a state of quiescence in the dry seed to a state of highly active metabolism and terminates with embryonic axis elongation (Bewley and Black, 1994). Generally, seed germination can be divided into three distinct stages from I to III (Bewley, 1997). Phase I includes a rapid initial uptake in which seeds are fully inflated, the structures and enzymes necessary for initial germination are present, and storage substances, such as starch, proteins, and lipids, that provide nutrition and energy for seed germination, begin to be activated. Phase II emerges immediately after Phase I, and is a plateau phase in which metabolism and cellular activity increase quickly, grain morphological and structural changes are obvious, the radicle and germ appear, and enzymes involved in various physiological processes and morphogenesis are abundantly expressed. In Phase III, also called postgermination, seed protrusion emerges, water uptake increases, plants absorb moisture from the surroundings, radicle cells continue to elongate, cell division and DNA synthesis are accelerated, and large amounts of stored reserves are mobilized. After seed protrusion, ATP content increases rapidly and energy is supplied from the endosperm to simultaneous external and endosperm support (Hourmant and Pradet, 1981).

Transcriptome investigations during seed germination have been performed in a number of cereal crops, such as wheat (Yu et al., 2014), rice (Howell et al., 2009), maize (Jiménez-López et al., 2011), and barley (Sreenivasulu et al., 2008). However, gene function is ultimately determined by the protein product, and therefore exploring the dynamic changes during seed germination at the proteome level is vital to gain an in-depth understanding of the physiological and biochemical characteristics of seed germination. Bread wheat (*T. aestivum* L., AABBDD) is a hexaploid species with a huge genome (up to 17,000 Mb; Brenchley et al., 2012). Previous proteomic studies on wheat germination mainly aimed to reveal the molecular mechanism of germination (Ahmad et al., 1998), and determine the influence of the external environment (Tucaliuc et al., 2008), specific protein changes (de Gara et al., 1997), and different organs (Mak et al., 2009; He et al., 2015). The proteomics is a state-of-the-art approach for discovering the molecular mechanisms upon seed germination, and have applied to the studies of seed germination in response to environment changes (He and Yang, 2013; Tan et al., 2013). Recent advances in genome projects on the A^u^ genome of *Triticum urartu* (Ling et al., 2013), D^t^ genome of *Aegilops tauschii* (Jia et al., 2013), and the whole genome in hexaploid wheat [Brenchley et al., 2012; Choulet et al., 2014; The International Wheat Genome Sequencing Consortium (IWGSC), 2014], would provide great possibility for proteomics study. In addition, protein phosphorylation is an important posttranslational modification that controls a wide range of biological processes and occurs in all eukaryotic subcellular compartments (Sugiyama et al., 2008). Previous studies have indicated that starch phosphorylation affects the rate of starch decomposition (Edner et al., 2007). A method for labeling wheat callus with [^32^P]orthophosphate *in vivo* was used to identify several phosphoproteins that took part in protein synthesis (Rampitsch et al., 2006). Several signaling proteins such as GTP-binding proteins, 14-3-3 proteins, and calcium-binding proteins were detected in the signaling pathway between wheat and the fungal pathogen *Septoria triticin* (Yang et al., 2013). Fifty-two phosphorylated transcription factors (TFs), 85 protein kinases (PKs), and 16 protein phosphatases (PPs) were shown in the study of bread wheat seedling leaves (Lv et al., 2014). Sixty-one phosphoproteins showed significant changes in phosphorylation level of the developing grains under well-watered and water deficit conditions according to the study of Zhang et al. (2014). However, the roles of protein phosphorylation in seed germination are still unclear.

The Chinese bread wheat cultivar Jimai 20, released in 2003 and widely cultivated at present in main wheat production areas of China, has many outstanding characteristics, such as strong tillering, higher panicle rate, improved drought stress tolerance, good lodging resistance, high yield and genetic stability, and strong adaptability, as well as superior dough quality (Luo et al., 2006; Liu et al., 2009). In this study, we performed the first dynamic proteome analysis during seed germination of Jimai 20 by a two-dimensional differential gel electrophoresis (2D-DIGE)-based proteomic approach. Some key phosphorylated proteins involved in seed germination were detected and identified by Pro-Q Diamond and liquid chromatography-tandem mass spectrometry (LC-MS/MS). Our results provide new insights into the biochemical mechanisms underlying wheat seed germination.

## Materials and methods

### Plant materials and germination treatment

Elite Chinese bread wheat cultivar (*T. aestivum* L.) Jimai 20 was used in this study. Seeds with similar size and weight were selected, and were washed with distilled water three times. Wheat germination was triggered by seed imbibition, according to seed imbibition time and morphological characteristics. Seed samples with three biological replicates were collected at 0 h after imbibition (HAI, dry seeds), 12 HAI (seeds were soaked in water for 12 h), 24 HAI (radicles were about to emerge), 36 HAI (radicles come up to half of seed), and 48 HAI (germs become green). Collected samples were stored at −80°C prior to analysis.

### Scanning electron microscopy (SEM) observation

SEM analysis was performed based on previous method (Dong et al., 2012). The seeds were cut in half transverse which were parallel to ventral side, and they were observed with a SEM S-4800 FESEM (Hitachi, Japan).

### Protein preparation and 2D-DIGE

Seed proteins were extracted as following procedures. Seeds of 500 mg were ground to fine powder in extraction buffer (0.25 M sucrose, 0.02 M Tris–HCl pH 7.5, 0.01 M EGTA, 0.001 M PMSF, 4% triton X-100) for 10 min using a mortar and pestle. Proteins were extracted through vortex for 15 min at room temperature. After centrifuging at 13,000 rpm for 10 min in 4°C, supernatants were transferred to new tubes, and five-fold volumes of 0.1 M ammonium acetate-methanol were added to supernatant for 2 h at −20°C, and then the pellets were centrifuged at 13,000 rpm for 15 min. The pellets were rinsed with 500 μl cold (−20°C) acetone including 1% v/v β-mercaptoethanol, followed by centrifuging at 13,000 rpm for 5 min. This step was repeated three times. After freeze drying, protein samples were collected and stored at −80°C. Protein powders were redissolved by lysis buffer containing 7 M urea, 2 Mthiourea, and 4% w/v CHAPS. Protein concentrations were determined with a 2D-Quant-Kit (GE Healthcare, USA), and then used for two-dimensional gel electrophoresis (2-DE) analysis.

Protein separation consists of protein labeling and 2D-DIGE based on the published methods (Dong et al., 2012), and the details of 2D-DIGE experiments were showed in Table S1. IEF was performed with the Ettan IPG-phor system (GE Healthcare, USA) in immobiline pH gradient DryStrips (GE Healthcare, pH 3–10, 18 cm, USA). Proteins of 600 μg extracted from each sample (three biological replicates) were run separately on conventional 2-DE gels for protein identifications. The gels were stained with colloidal Coomassie Brilliant blue G-250 (CBB; Sigma, USA).

### Image analysis and protein identification through mass spectrometry

The gel image analysis was performed by following Gao et al. (2009). Protein spots with statistically significant two-fold or greater differences in abundance between samples were determined with Student's *t*-test (*p* < 0.05). Differentially expressed protein (DEP) spots were excised from gels, and identified through matrix-assisted laser desorption/ionizationtime-of-flight mass spectrometry (MALDI-TOF-MS) and tandem mass spectrometry (MALDI-TOF/TOF-MS). The in-gel protein digestion steps and criterion were followed by previous methods (Gao et al., 2009; Dong et al., 2012). Tryptic peptides were analyzed with a MALDI-TOF mass spectrometer (SM, Shimadzu Biotech, Kyoto, Japan). Subsequent identifications were performed using a MALDI-TOF/TOF mass spectrometer 4800 Proteomics Analyzer (Applied Biosystems, Framingham, MA, USA). Spectra were acquired in a positive reflectron mode. The calibration mixtures (Applied Biosystems) were used to calibrate the spectrum to a mass tolerance within 0.1 Da. The parameters for database searching were peptide tolerance of 0.2 Da; MS/MS tolerance of 0.3 Da; one missed cleavages; variable modifications of Carbamidomethyl (Cys), oxidation (Met). GPS Explorer™ software version 2.0 (Applied Biosystem) was used to create and search files against the NCBI non-redundant green plant database. All the proteins identified have MASCOT report total ion scores >42 and identification probabilities of more than 95%. The mass spectrometry proteomics data have been deposited to the ProteomeXchange Consortium (Vizcaíno et al., 2014) via the PRIDE partner repository with the dataset identifier PXD003083.

### Detection and identification of phosphorylated proteins

After 2-DE separation as described above, the gels were stained with Pro-Q Diamond (GE Healthcare, USA) for phosphoprotein detection according to Agrawal and Thelen (2005). All staining and washing steps were performed on vortex (Forma Scientific 4520, USA) at 50 rpm. Gels stained by Pro-Q Diamond were imaged by using a 532 nm excitation laser and a 580 nm long pass filter on a Typhoon™ 9400 scanner (GE Healthcare, USA). Phosphorylated protein spots detected by Pro-Q Diamond were selected and collected from gels. After digested (Gao et al., 2009), they were further identified by liquid chromatography coupled to tandem mass spectrometry (LC-MS/MS) according to our recently established method (Cao et al., 2015). For MS analyses, peptides were resuspended in 0.1% FA and analyzed by LTQ Orbitrap Elite mass spectrometer (Thermo Fisher Scientific) coupled online to an Easy-nLC 1000 (Thermo Fisher Scientific) in the data-dependent mode. The sample was trapped on a 150 μm × 0.5 mm precolumn and eluted to an analytical 75 μm × 150 mm column. Peptides were separated by a linear gradient formed by 2% ACN, 0.1% FA (mobile phase A), and 98% ACN, 0.1% FA (mobile phase B), from 3 to 30% of mobile phase B in 90 min. The mass spectrometer was operated with full scan acquisition in the Orbitrap at 24,000 resolutions (350–1800 m/z). The mass spectrometer was operated acquiring CID MS/MS scans after each MS1 scan on the 25 most abundant ions with MSA central losses of m/z 98, 49, and 32.6. The MS raw data were analyzed using the MaxQuant software version 1.3.0.5 and searched against the NCBI_triticum database. For searching, the enzyme specificity was set to trypsin with the maximum number of missed cleavages set to 2. Oxidized methionines, phosphorylation addition to serine, threonine, and tyrosine, and N-terminal protein acetylation were searched as variable modifications. Carbamidomethylation of cysteines was searched as a fixed modification. The false discovery rate (FDR) for peptide, protein, and site identification was set to 1%. The CID normalized collision energy was set to 35. The phosphorylation sites and localization were determined based on recent reports (Zhang et al., 2014; Hao et al., 2015).

### Bioinformatics analysis

Hierarchical cluster analysis of the DEP spots was performed according to Valledor et al. (2010). For pathway analysis, the proteins were firstly searched the database of *B. distachyon, O. sativa* and *A. thaliana* in National Center for Biotechnology Information (NCBI) database, matching proteins with similarity maximum, and then searched the KEGG *B. distachyon, O. sativa* and *A. thaliana* data base and mapped to the *T. aestivum* specific pathways with KEGG Mapper. Analysis of protein-protein interaction (PPI) was followed by three steps: (1) Protein sequences of all DEPs were obtained through BLAST analysis with the NCBI database, and protein eukaryotic orthologous group (KOG) numbers were matched in the KOG database; (2) All KOG numbers were collected for PPI analysis using STRING database (version 9.1, http://string-db.org) (Franceschini et al., 2013), and (3) The network with a confidence score of at least 0.700 was constructed, and displayed using Cytoscape (version 3.0.2) software (Cline et al., 2007). According the sequences of certain phosphoproteins, the occurring of homologous protein phosphorylation in different species was searched in Plant Protein Phosphorylation Data Base (P^3^DB, http://www.p3db.org/index.php). Prediction of phosphorylated modification sites was done in NetPhos (version 2.0, http://www.cbs.dtu.dk/services/NetPhos/) (Blom et al., 1999) based on protein sequences. 3D structure prediction of the interesting phosphoproteins was performed by Phyre2 (the protein homology/analogy recognition engine v 2.0, http://www.sbg.bio.ic.ac.uk/phyre2/html/page.cgi?id=index), and the exhibition of 3D structures were utilized Swiss-PdbViewer (SPDBV) software (version 4.1, http://spdbv.vital-it.ch/).

## Results

### Seed morphological and structural changes during germination

**Figure 1:**
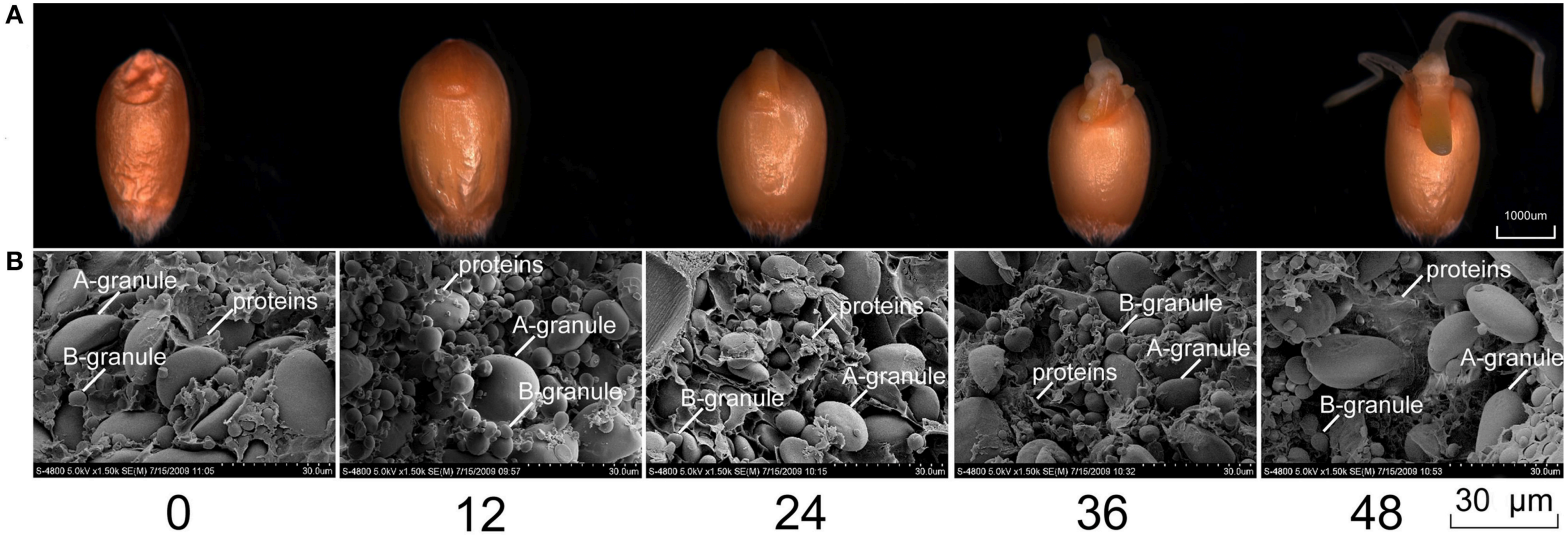
**Grain morphological changes (A) and SEM profiles (B) of five seed germination stages in bread wheat cultivar Jimai 20**.

Seed germination began with seed rehydration, as shown in Figure 1A. Upon imbibition, wheat seeds were fully inflated at 12 HAI (hours after imbibition). The radicle emerged at 24 HAI, and grew approximately half the length of the grain until 36 HAI; the germ and two lateral roots began to appear simultaneously. After 48 HAI, the germ began to turn green, indicating the imminent commencement of photosynthesis and that nutrition provision for seed germination will change from endosperm to simultaneous external and endosperm support.

The mature seeds (0 HAI) showed accumulation of large amounts of starch granules and storage proteins (Figure 1B). At 12 HAI, starch granules began to expand, and storage proteins were partially hydrolyzed to water-soluble proteins, and further degraded into amino acids by peptide hydrolases (Bewley and Black, 1994). With an extended germination time, the volume of storage proteins inside grains decreased after 36 HAI, suggesting that various physiological and biochemical processes occurred during this period, and cellular activity was relatively abundant. However, the changes in starch granules at the end of germination were not obvious in either number or morphological appearance, which may be related to the shorter germination time. At 48 HAI, seeds did not enter into postgermination, and most of the storage substances were not mobilized. A previous study showed that large-scale storage substance mobilization did not begin before postgermination (Bewley, 1997).

Phase I was initialized upon imbibition and terminated with radicle emergence (Bewley, 1997). According to our observations, the periods from 0 to 24 HAI, 24 to 48 HAI, and after 48 HAI generally correspond to Phase I, II, and III, respectively.

### DEP identification and functional classification

**Figure 2:**
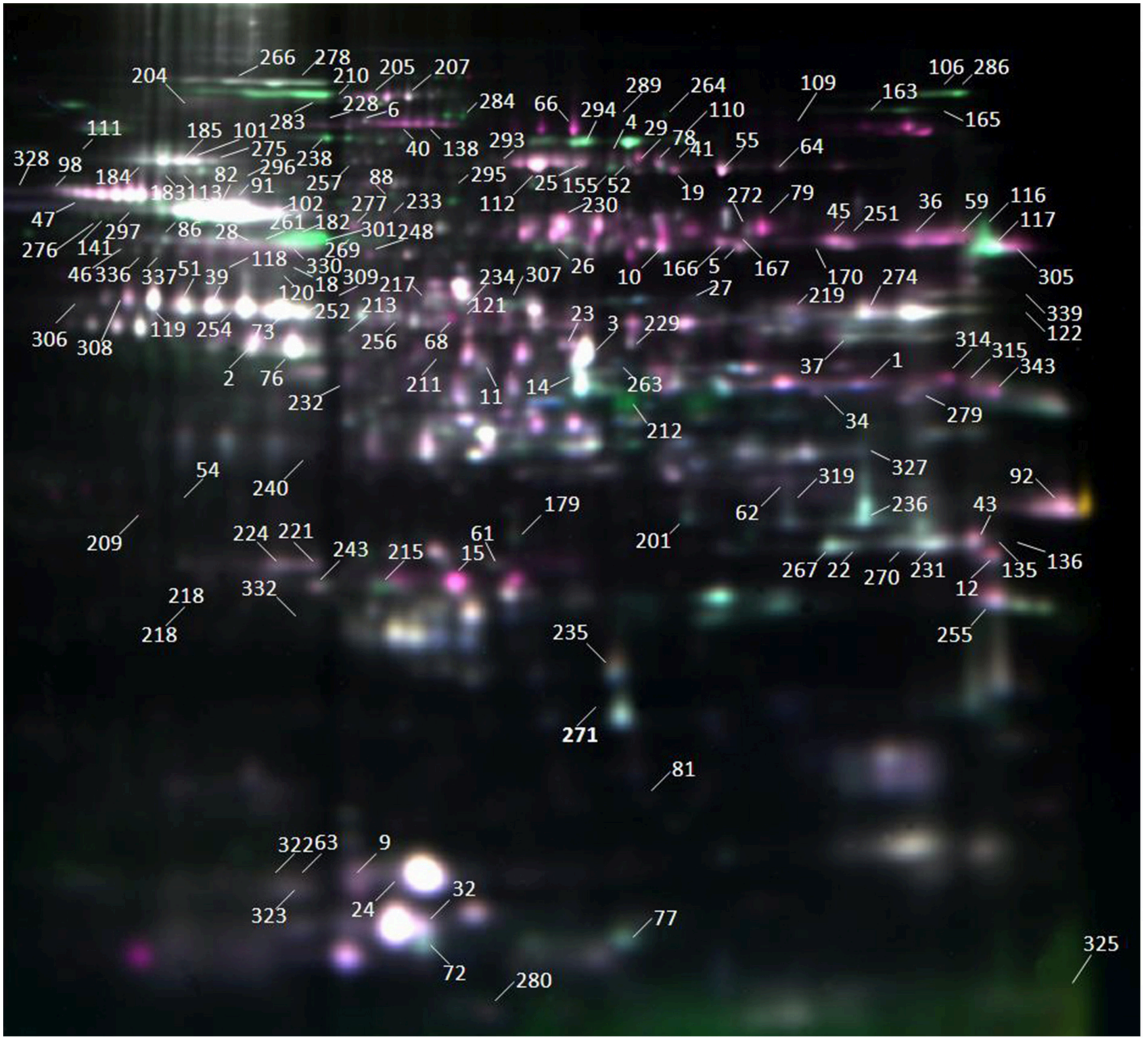
**Images from 2D-DIGE analysis of whole proteins in seed germination of Jimai 20**. This gel was Gel 1 as shown in Table S1. Samples were electrofocused on a 18 cm pH 3–10 linear IPG strip and separated by SDS-PAGE performed on 12% polyacrylamide gels. DEP spots were numbered on gel image.

DEPs during the five seed germination stages were detected by 2D-DIGE (Figure 2), and conventional 2-DE images are presented in Figure S1. Among 166 protein spots with at least two-fold differences in abundance, 88 and 78 spots were identified by MALDI-TOF-MS and MALDI-TOF/TOF-MS, respectively. These proteins represented 73 unique proteins (Table S2), and their peptide data are presented in Table S3.

**Figure 3:**
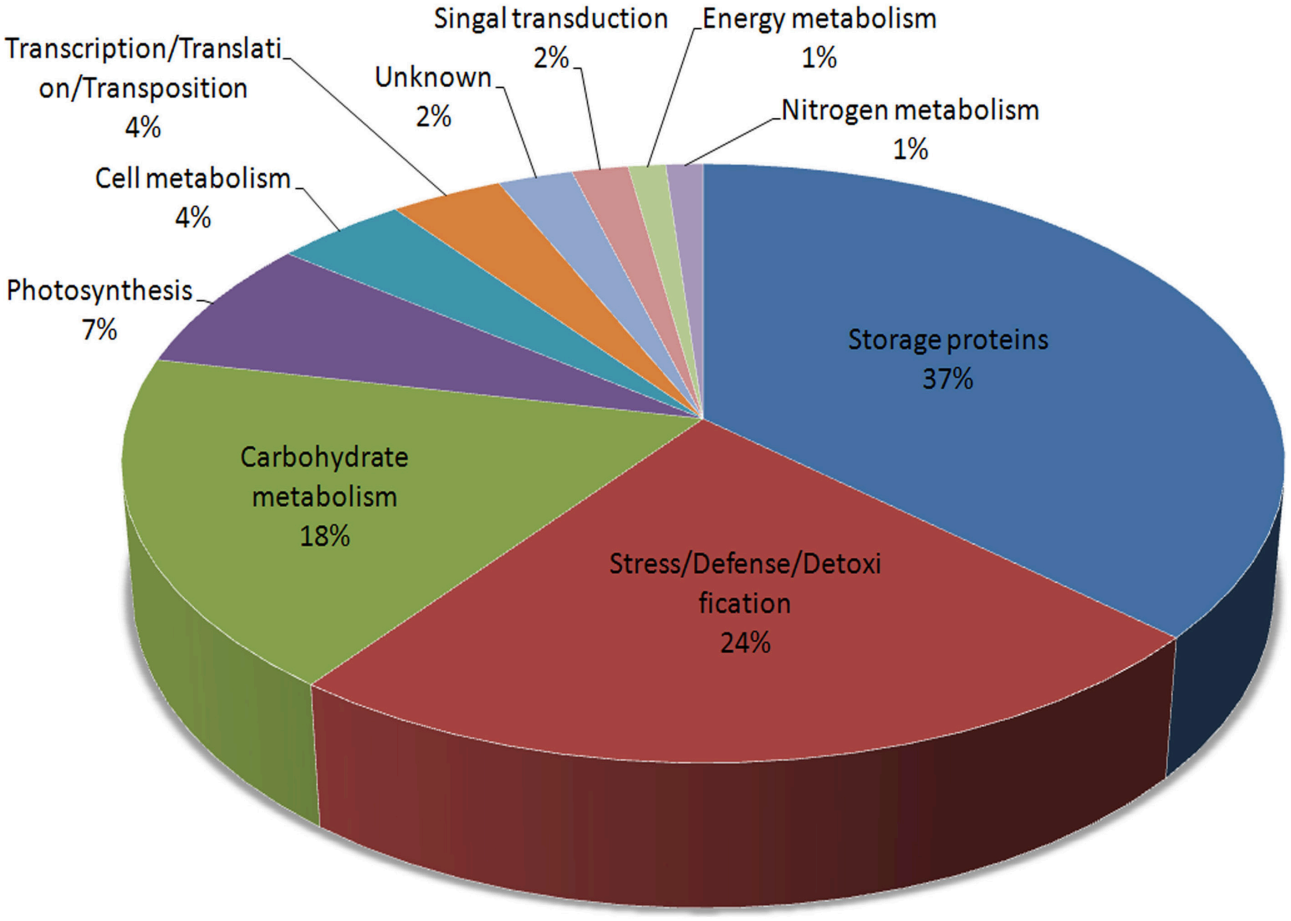
**Functional distributions of the identified DEPs during grain germination in Jimai 20**.

These 73 unique proteins belong to 10 functional categories (Figure 3): storage proteins (36.75%), stress/defense/detoxification (23.49%), carbohydrate metabolism (18.07%), photosynthesis (7.23%), cell metabolism (4.22%), transcription/translation/transposition (3.61%), signal transduction (1.81%), nitrogen metabolism (1.2%), energy metabolism (1.2%), and unknown (2.41%). Furthermore, proteins related to carbohydrate metabolism were divided into four subcategories: alcoholic fermentation, glycolysis/Calvin cycle reactions, carbon skeleton, and starch synthesis/degradation. Storage proteins included globulins, gliadins, glutenins, and other storage proteins. Those related to cell metabolism were grouped into cell division and cell wall synthesis categories (Table S2).

### Hierarchical cluster analysis of DEP expression

**Figure 4:**
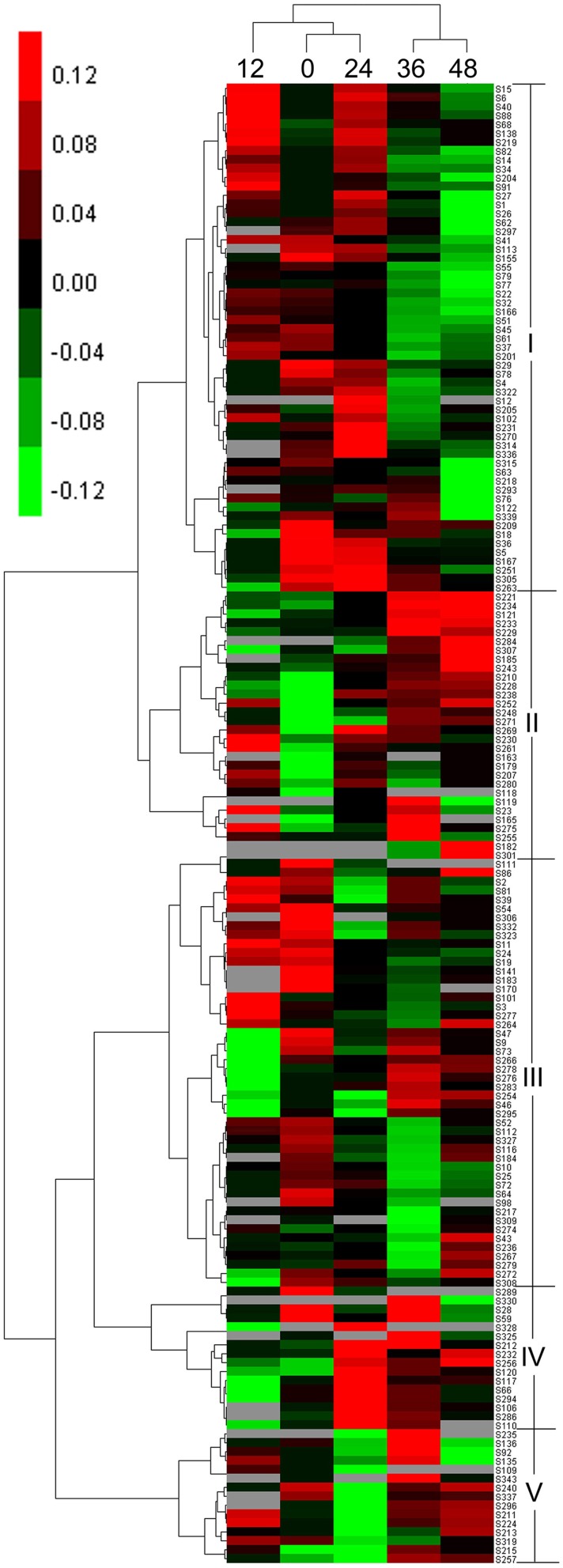
**Hierarchical clustering analysis of DEP spots**.

According to the hierarchical cluster analysis and percent (%) volume values listed in Table S4, the dynamic expression profiles of the identified DEP spots at five seed germination stages were determined and are shown in Figure 4. There were two distinct clusters corresponding to the first three stages (0, 12, and 24 HAI) and the last two stages (36 and 48 HAI), respectively. Compared with 12 HAI, 0 and 24 HAI showed greater similarities in expression patterns because there was higher seed moisture content at 12 HAI and many enzymes involved in stored substance mobilization were maintained (Bewley, 1997). Similar expression profiles were also found at 36 and 48 HAI.

Among 166 DEP spots separated into two clusters, five distinct expression patterns were found during seed germination, designated as patterns I–V. The first cluster containing pattern I and pattern II displayed upregulated expression first after imbibition. Pattern I, with 57 DEP spots, contained large amounts of storage proteins and generally showed an up-down expression trend, with higher levels of expression emerging at 12 HAI, in proteins mainly associated with storage substance degradation, cell division, cell wall synthesis, energy metabolism, and signal transduction. Pattern II had 30 DEP spots and displayed an upregulated expression trend. This group mainly included enzymes related to energy synthesis, starch degradation, and small molecules synthesis, such as glycolysis and amino acid generation. The other three expression patterns (III–V) were grouped into another cluster. Pattern III, with 48 DEP spots, exhibited a down-up expression trend, in which some DEP spots were downregulated at 0–24 HAI and upregulated at 36 or 48 HAI, and mainly include storage proteins and enzymes related to starch degradation, cell wall synthesis, energy metabolism, and ribulose-1,5-bisphosphate carboxylase/oxygenases (RuBisCO). Pattern IV contained 16 DEP spots and showed a down-up-down expression trend. Their expression peak emerged at 24 HAI and involved many functional groups, such as RuBisCO, starch degradation, and alcoholic fermentation. These DEPs mainly play roles in transformation from Phase I to II, and are useful for tissue and organ generation. Similar to pattern IV, pattern V, including 15 DEP spots, also showed a down-up-down expression trend, but their expression peak mainly occurred at 36 HAI. This group included different enzymes related to storage, RuBisCO, small molecule metabolites, etc.

### Bioinformatics based protein–protein interaction network analysis of key DEPs involved in seed germination

**Figure 5:**
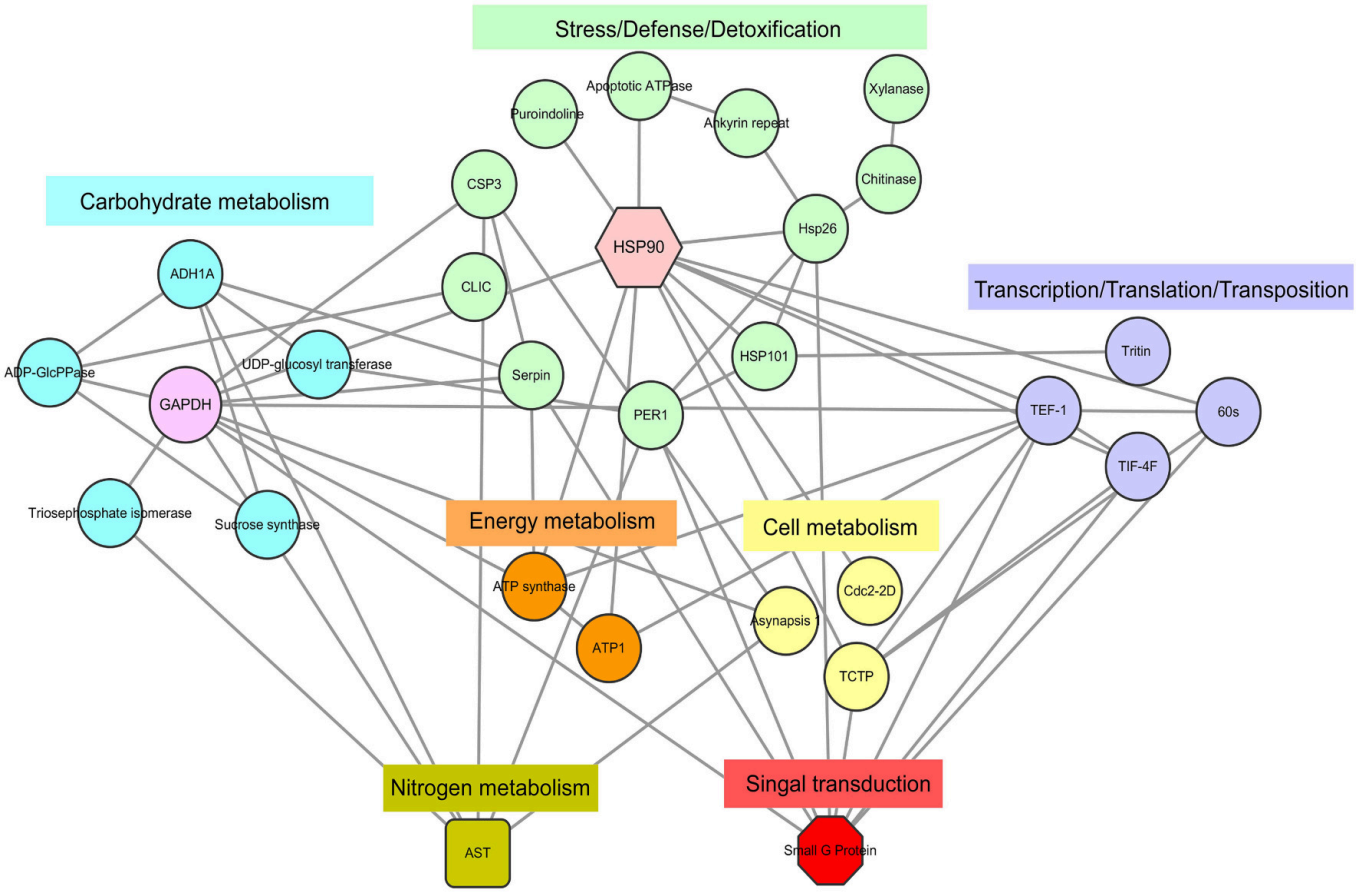
**DEPs regulatory network centered by HSP 90 and GADPH**. Interactions of the DEPs were extracted by searching the STRING database with a confidence cutoff of 0.700. The interaction network was reconstructed by using the Cytoscape software. The details of all the protein nodes were listed in Table S5.

Figure 5 shows the results from the protein–protein interaction network analysis, which indicated seven important functional categories with a total of 29 KOGs (Table S5) mainly involved in carbohydrate, nitrogen, cell and energy metabolism, signal transduction, transcription/translation/transposition, and stress/defense/detoxification. The groups of carbohydrate metabolism and stress/defense/detoxification included more members, concentrated as glyceraldehyde-3-phosphate dehydrogenase (GAPDH) and heat shock protein (HSP) 90, respectively. With the onset of seed germination, abundant proteins involved in carbon metabolism appeared, such as glyceraldehyde 3-phosphate dehydrogenase (GADPH), alcohol dehydrogenase (ADH), and sucrose synthase (SS). Combined with proteins related to energy metabolism, the large amounts of energy needed for germination were produced. When germination entered Phase II, cellular activity increased, and signal transduction, nitrogen metabolism, protein transcription, and translation became active. Due to environmental changes, many proteins that participate in defense stress were expressed. Several signal molecules could mediate some plant defense mechanisms. The above results indicate that all of these physiological groups interact with each other, suggesting that various metabolic processes may act synergistically during seed germination.

### Phosphorylated protein characterization during seed germination

As shown by 2D-DIGE (Figure 2), many protein spots with the same molecular weight but different isoelectric points or vice versa were identified as the same proteins, such as SS type I (spots 6 and 228), serpin (spots 39, 51, and 232), and beta-amylase (BAM, spots 18, 47, 82, 86, 88, 91, 98, 102, 141, 182, 209, 276, 297, 301, 328, and 330). These isoforms were probably due to protein phosphorylation. Upon Pro-Q Diamond staining for phosphorylation (Figure S2), some proteins showed strong staining signals, indicating that these protein spots were indeed phosphorylated. To further identify these proteins, stained spots were collected and identified by MALDI-TOF/TOF-MS. Peptide data are listed in Table S6. These phosphoproteins identified by MALDI-TOF/TOF-MS may not be phosphorylated proteins because one spot may contain more than one protein. Thus, they are putative phosphoproteins. In total, 14 protein spots representing five putative phosphorylated proteins were identified, i.e., globulin 3 (spots 5, 9, 26, 240, and 323), SS type I (spot 6), serpin (spot 39 and 232), BAM, partial (spot 82, 98, and 141), BAM (spot 102 and 301), and plastid ADP-glucose pyrophosphorylase (AGPase) small subunit (spot 269). These proteins are mainly involved in storage substance degradation and plant defense.

The potential modification sites of five identified putative phosphorylated proteins were predicted using NetPhos (Figure S3); all of these proteins included potential phosphorylated modification sites. The sequence alignment performed in the P^3^DB database is shown in Figure S4. In previous studies, all of these proteins except for globulin 3 were shown to have phosphorylation sites in multiple species, including *Oryza sativa, Medicago truncatula, Vitis vinifera*, and *Arabidopsis thaliana*. Sequence alignment indicated that these modification sites in wheat were conserved with other species, such as sites 10 and 149 of SS, site 204 of serpin, and site 225 of AGPase. The phosphorylated protein information searched in the P^3^DB database is listed in Table S7.

**Figure 6:**
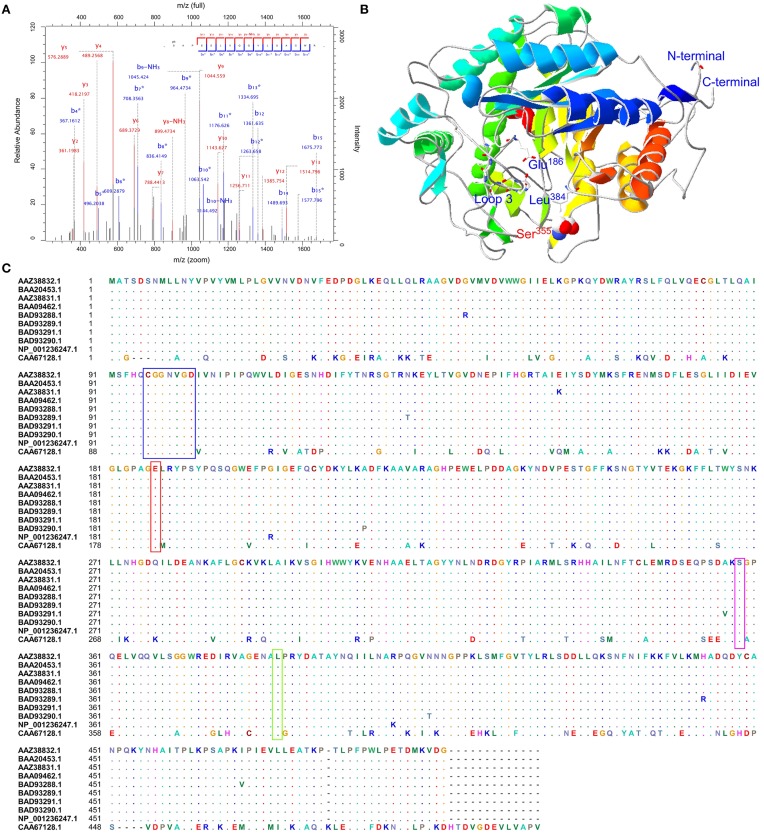
**Sequence alignment, three-dimensional structure and LC-MS/MS spectra map for BAM**. **(A)** Phosphopeptide LC-MS/MS spectra map; **(B)** Three-dimensional structure of BAM; **(C)** Sequence alignment with *T. aestivum* and *G. max*. Red box showed a conserved residue. Glu^186^: the catalytic center; Green box presented Leu^384^ residues, and participated in binding the outer chains; Blue box was a peptide forming loop 3, which was helpful to make BAM extend into solvent.

To confirm these putative phosphorylated proteins and determine the locations of phosphorylation sites, all 14 protein spots were identified by LC-MS/MS. Due to the sensitivity of instrument and the limitation of the database, sometimes, few proteins may be identified through the LC-MS/MS. This phenomenon was not unique in this study. In our recent study, Chen et al. (2014) also only identified few phosphosites for starch synthesis related protein GBSSI from one band (Chen et al., 2014). This study showed that BAM (gi|1771782, CAA67128.1, spot 301) contained one serine phosphorylation site at Ser^355^ (_S(ph)APEELVQQVLSAGWR_) (Figure S5). The phosphopeptide LC-MS/MS spectra, the predicted 3D structure, and phosphorylation site conservation of BAM are shown in Figures 6A–C, respectively. The crystal structure of soybean (*Glycine max*) BAM has been reported (Mikami et al., 1993), and its catalytic center was shown to be located near the carboxyl group of Glu^186^ residues and the methyl groups of Leu^384^ residues participating in the binding of the outer chains of starch. In addition, residues 96–102 of loop 3 could form a significant tertiary structure, which contributes to its extension in solvent (Mikami et al., 1993). Based on sequence alignment with *T. aestivum* and *G. max* (Figure 6C), the amino acid sequences of BAM are highly conserved. Combined with the predicted 3D structure of BAM (Figure 6B), similar to soybean, this enzyme in wheat was also predicted to form a core utilizing the (α∕β)_8_ supersecondary structure, and Glu^186^, Leu^384^, loop 3, and the phosphorylation site, Ser^355^, were all located in this core.

Various phosphoproteins appeared to be phosphorylated during different periods (Figure S6). Phosphorylation of globulin 3 mainly occurred at 12 HAI. Spots 9 and 323 may represent degradation products of globulin 3. BAM, AGPase, and SS, all involved in storage substance degradation, accumulated during grain maturation, and were activated through phosphorylation (Subbaiah and Sachs, 2001; Kötting et al., 2010). Highly phosphorylated BAM and AGPase appeared during Phase I, which is likely related to storage decomposition after imbibition. SS had a high phosphorylation level during Phase II, which may be due to its involvement in preparation for stored reserve mobilization during postgermination.

## Discussion

Upon imbibition, numerous storage proteins, starch, and sugars were mobilized during Phase I, and the dry seeds transited rapidly from a quiescent state to a metabolically active state. Cell structures, such as membranes, were disturbed because of imbibition, leading to rapid leakage of solutes and low molecular weight metabolites into the surrounding environment. In addition, respiratory activity showed a steep initial increase in Phase I, and declined until the radicle emerged (Bewley, 1997), so those enzymes involved in energy metabolism showed high levels of expression in this phase. With the exception of substance and energy metabolism during Phase II, large amounts of RuBisCO participating in CO_2_ fixation were detected, which would aid in the development of vegetative organs. Simultaneously, CO_2_ fixation was a prelude of photosynthesis, and the high expression levels of these proteins heralded the germination transformation from Phase II to III. At 48 HAI, the germ began to turn green, Phase II started to transit to Phase III, and plant nutrient supply transitioned from seed supply to a combination of seed and environmental supply. Hence, the downregulation of these proteins at 48 HAI may be relevant to conversion of nutrient supply.

In addition, these DEPs involved in stress/defense/detoxification showed different expression profiles, which were related to various metabolites and environmental conditions during the different germination periods. Due to the desiccation process in grain maturation (Finnie et al., 2002), various proteins with roles in defense against desiccation were accumulated. Thus, upon imbibition, the moisture content increased and the seed defense mechanism was activated.

Apart from that, we further identified one phosphorylated protein beta-amylase involved in starch degradation by Pro-Q staining and LC-MS/MS. Although many protein spots on 2-D gel can be stained by Pro-Q, a few of them were confirmed to be phospholated (Figure S5). The possible reasons may be the huge genome of allohexaploid wheat (up to 17 Gb) that generally results in multiple proteins included in one spot on the 2-D gel. This would affect the results of Pro-Q staining and LC-MS/MS identification.

### DEPs involved in storage substance mobilization and energy supply

The main physiological characteristics of the three phases during seed germination were storage degradation, physiological processes/morphogenesis, and photosynthesis (Bewley, 1997). In Phase I, proteins involved in storage substance mobilization and energy generation were highly expressed. Upon imbibition, storage substances began to decompose, BAM (spots 18, 47, 82, 86, 88, 91, 98, 102, 141, 182, 209, 276, 297, 301, 328, and 330) and SS (spots 6, 40, 138, and 228) converted starch into UDP-glucose and fructose, which are important for the metabolic, structural, and storage functions of the plant cell (Sturm and Tang, 1999; Fox, 2009). Meanwhile, phosphorylation of globulin 3 occurred at 12 HAI. Previous studies on 12 S globulins in *Arabidopsis* indicated that phosphorylation participated in protein processing, assembly, and activation (Wan et al., 2007). BAM and AGPase are mainly involved in breaking down macromolecular substances, but SS participates in small molecule degradation, and therefore high levels of BAM and AGPase phosphorylation occurred at Phase I, and SS emerged at Phase II (Mikami et al., 1993; Edner et al., 2007).

On the other hand, the main functions of central carbon metabolism (glycolysis, TCA cycle) during germination provide the energy needed for subsequent plant growth and development. Simultaneously, carbon metabolism could be an effective connection with other metabolic processes (Nicolás and Aldasoro, 1979). GADPH (spots 3 and 11) and triosephosphate isomerase (spots 221 and 224), together with transketolase, are conducive to triosephosphate synthesis, which could be used in energy production and carbon skeleton synthesis (Bohler et al., 2013). Phosphoenolpyruvate carboxylase (PEPC, spot 46) produces oxaloacetate, which is then converted to malate that replenishes the citric acid cycle, providing the necessary carbon skeletons for nitrogen assimilation (Huppe and Turpin, 1994), which is important to connect carbon and nitrogen metabolism. Due to the gaseous diffusion restrictions between the seed and the surrounding environment, the seed interior maintains a state of oxygen deficiency, and frequently produces ethanol in the initial stages of germination (Morohashi and Shimokoriyama, 1972). ADH (spots 68 and 256), the key enzyme in primary short-chain alcohol metabolism (Brändén et al., 1975), participates in anaerobic glycolysis, producing ATP under conditions of oxygen deprivation (Freeling and Benett, 1985). ADH showed higher expression at 12 HAI or 24 HAI (Table S2), which could be helpful for maintenance of glycolysis at a stable level. In addition, sufficient enzymes involved in the TCA cycle and terminal oxidases were accumulated during the seed maturation process. These enzymes stored in the mitochondria in dry seeds are activated rapidly after imbibition, which provides adequate amounts of ATP to support metabolism in the early stages of germination (Ehrenshaft and Brambl, 1990; Attucci et al., 1991). Among them, the highest levels of ATP synthase beta subunit (spot 261) were seen at 12 HAI (Table S2) in our study, which could be beneficial for catalyzing the formation of ATP (Mills and Richter, 1991; Ducos et al., 2001). These observations indicate that energy metabolism is activated after imbibition and energy supply is critical for seed germination.

### DEPs related to physiological metabolism and organizational development

Phase II began at radicle emergence and terminated with the germ turning green, called a plateau phase (Bewley, 1997). Various physiological metabolic pathways and organizational development became more active, and marked cell activities, protein translation, and transport occurred during this phase. First, amino acid synthesis is crucial for protein translation. Aspartate amino transferase (AST, spot 23 and 229) catalyzes the synthesis of aspartate, and aspartic acid as the starting material participates in the synthesis of various amino acids, including four essential amino acids (Good and Muench, 1992). Protein translation is essential for organizational development in seed germination. In this study, the radicle and germ began to germinate at 12 and 36 HAI, respectively, which corresponded to the peaks of AST expression (Table S2).

Substance transportation-related protein UGT (spot 27), which is localized to the endoplasmic reticulum membrane, is capable of transferring glucuronic acid, and is useful for molecular efflux (Mentewab et al., 2005). In the present study, this protein showed the highest expression level at 36 HAI, suggesting that the transportation of abundant small molecules decomposed in Phase I was activated during Phase II. Along with radicle development, active cellular activity appeared, and cell division, elongation, and cell wall synthesis contributed to tissue development. Cdc2 (cdc-2D, spot 106), designated as cyclin-dependent protein kinase, drives the cell into mitosis (Nurse, 1990), and asynapsis 1 (ASY1, spot 293) involved in meiosis I, promotes chromosome synapsis and homologous chromosome pairing (Boden et al., 2007, 2009). Our results indicate that high expression levels of both proteins occurred at 24 HAI, which would improve cell mitosis and metabolism during seed germination. Another key protein, translationally controlled tumor protein homolog (TCTP, spot 218), which controls cell size and number (Hsu et al., 2007), showed relatively stable expression, but a noticeable decrease was observed in the last stage, suggesting that cell division and growth became activated after imbibition. TCTP was downregulated at 48 HAI (Table S2), which may have been related to changes in nutrient supply (Mak et al., 2009).

### DEPs involved in photosynthesis in postgermination

When the germ began to turn green, the progress of germination entered Phase III, in which the nutrient supply mode began to transition from seed supply to the seed and surrounding environment, also called postgermination. In this phase, physiological metabolism became more active through water uptake, and stored reserves were completely exhausted with cell division and elongation, as well as radicle and germ growth (Bewley, 1997). However, the most notable feature of Phase III was the commencement of photosynthesis (Yu et al., 2014). Due to germination time constraints, postgermination was not the focus of this study, but large amounts of RuBisCO involved in dark reaction/photorespiration were found, including RuBisCO (spots 289 and 309), RuBisCO small subunit (spots 32, 81, 109, 201, 238, 252, 257, 264, and 322), and RuBisCO small chain precursor (spot 255). RuBisCO is a rate-limiting factor for both photosynthesis and photorespiration, is involved in CO_2_ fixation in photosynthesis, and in the production of 2-phosphoglycolate in the photorespiratory pathway (Evans, 1986; Makino et al., 1988). Thus, the accumulation of RuBisCO in Phase II contributed to the onset of photosynthesis.

### DEPs associated with stress response and defense

During seed germination, various defense proteins stored in mature dry seeds are activated by signals such as moisture, oxygen, temperature, superoxide ions, and abscisic acid (ABA; Durbin and Langston-Unkefer, 1988; Finnie et al., 2002). Several signaling proteins also play roles in plant defense mechanisms, such as ras-related GTP binding protein (spot 179); this is a DNA damage response protein, and may be involved in the translocation of signaling proteins to the nucleus (Aslam et al., 2010). Moreover, many proteins involved in responses to various stress conditions were identified in this study. In Phase I, along with changes in environmental conditions, such as temperature, water content, and oxygen concentration, proteins involved in responses to these abiotic stresses began to be expressed (Vierling, 1991; Tari and Csiszar, 2003), such as peroxidase 1 (spots 1, 34 and 279) and HSPs (spots 63, 204, 205, 207, and 230), with upregulation at 12 HAI (Table S2). During Phase II, with a decrease in water content, the radicle and germ began to develop, and alpha-amylase inhibitor (spots 235 and 271) was abundantly expressed, which protects plants against wounding (García-Olmedo et al., 1987) and is beneficial for organ development. On the other hand, seeds may be exposed to various microorganisms during germination, such as pathogenic bacteria and fungi. In the present study, several DEPs involved in biotic stresses were identified. For example, we found an upregulation during Phase I of resistance protein (spot 280), involved in the plant innate immune system, which functions in pathogen recognition (Krasileva et al., 2010), and peroxidase 1 (spots 1, 34, and 279) and putative RNA-directed RNA polymerase (spot 37), which are involved in the defense against pathogen penetration (Matzke et al., 1996; Cosio and Dunand, 2009). In addition, family 11 xylanase in complex with inhibitor (Xip-I, spot 236) was able to defense attacks from not only pathogen, but also bacteria and fungi (Payan et al., 2004). Class II chitinase (spots 22, 135, 136, 231, 267, and 270) and serpin (spots 39, 51, 73, 76, 119, 120, 213, 232, 254, 306, and 308) also function in inhibition of fungal growth (Mauch and Staehelin, 1989; Pekkarinen et al., 2007). Higher expression levels of these proteins appeared in Phase I, suggesting the presence of various microorganisms in the environment following imbibition. Moreover, previous studies have indicated that serpin phosphorylation leads to increased enzymatic activity, and further formation of the serpin–protease complex could prevent degradation of storage substances (Ma et al., 2014). In our phosphorylation experiments, high expression levels of serpin were seen at 0 HAI (Table S2), which is beneficial for seed storage. The high serpin phosphorylation level occurred in Phase I (Figure S6) and contributed to the defense against moisture variation upon imbibition.

### Overview of central metabolic pathways involved in wheat seed germination

**Figure 7:**
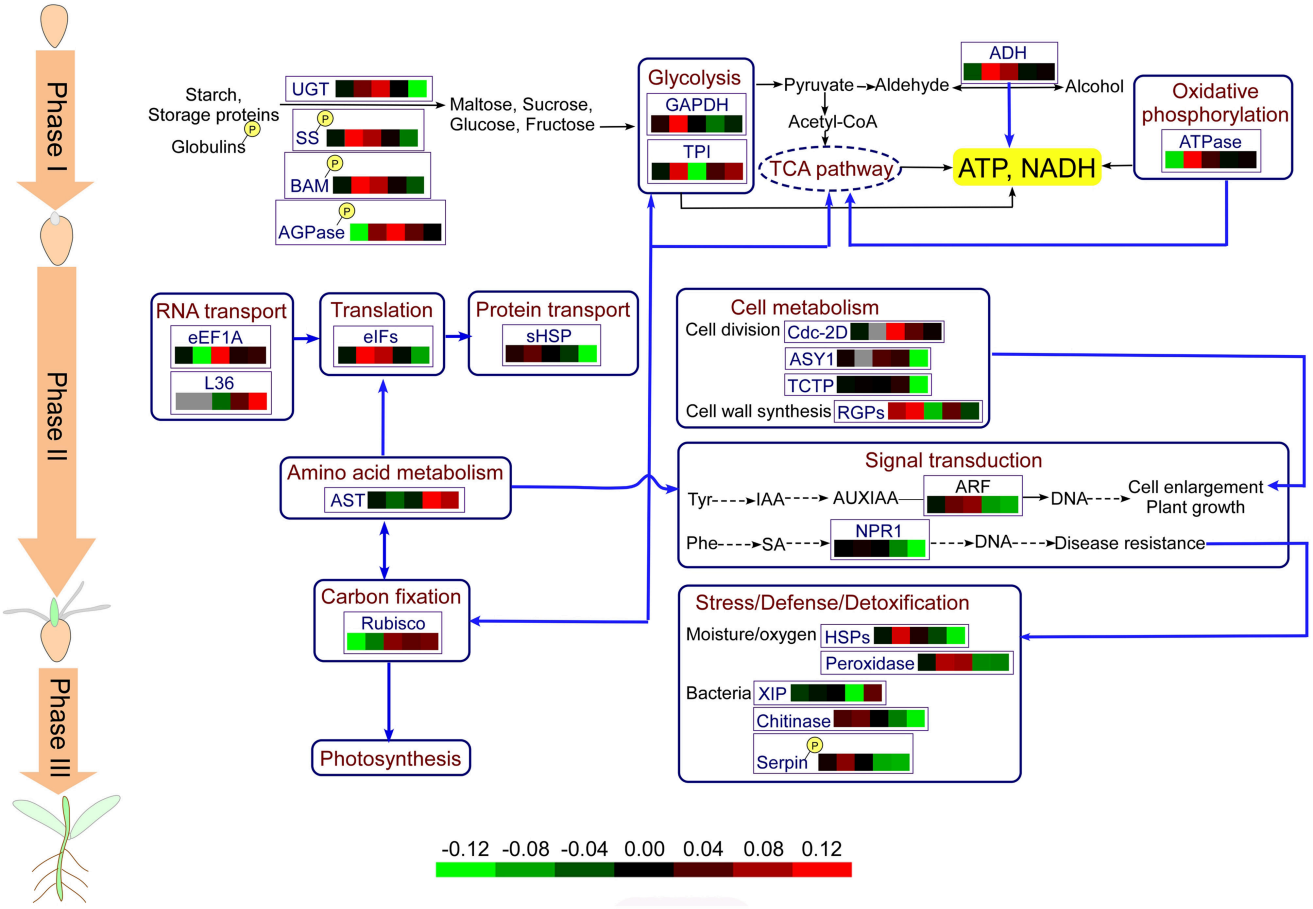
**Physiological metabolic pathways of identified DEPs**. The protein abbreviations with frames: AGPase, ADP-glucose-pyrophosphorylase; ADH, alcohol dehydrogenase; AST, aspartate aminotransferase; ASY1, asynapsis 1; ARF, auxin response factor; BAM, beta-amylase; eEF1A, elongation factor 1-alpha; eIFs, eukaryotic initiation factors; GAPDH, glyceraldehyde-3-phosphate dehydrogenase; RGPs, reversibly glycosylated polypeptides; HSPs, heat shock proteins; NPR, regulatory protein; Rubisco; ribulose-1,5-bisphosphate carboxylase/oxygenase; sHSP, small heat shock proteins; SS, sucrose synthase; TCTP, translationally-controlled tumor protein homolog; TCA, tricarboxylic acid; TPI, triosephosphat-isomerase; UGT, UDP-glucoronosyl/UDP-glucosyltransferase.

Based on the results obtained in this study and previous reports, we propose the central metabolic pathways involved in wheat seed germination in Figure 7. Seed germination begins with seed imbibition, which occurs in three distinct phases (I–III). In Phase I, upon rapid initial uptake, numerous storage substances and related degrading enzymes, including UGT, SS, BAM, and AGOase, begin to be activated, producing a small amount of energy. Phosphorylation of relevant enzymes plays an important role in these processes. The appearance of the radicle indicates transition of seed germination into Phase II, and due to grain morphological and structural changes, cell metabolism and cellular activity are highest in this stage. Various enzymes involved in protein synthesis and transport are highly expressed, and several amino acids, such as tyrosine and phenylalanine, which serve as signaling molecules, enter into the signal transduction pathways. Some plant defense mechanisms are mediated by signal transduction systems, and proteins involved in the plant stress system are activated by phosphorylation. In addition, amino acid metabolism is connected with CO_2_ fixation via oxaloacetate. CO_2_ fixation is vital for carbohydrate synthesis in the absence of O_2_ or under conditions of photosynthesis deficiency. Phase III, also called postgermination, begins with the germ turning green, indicating the onset of photosynthesis, and nutrient supply mode for seed germination transitions from seed supply to a combination of seed and environmental supply.

## Conclusions

In summary, this study revealed the first dynamic changes in the proteome involved in wheat seed germination by 2D-DIGE-based comparative analysis. Seed germination underwent three main stages: storage degradation, physiological processes/morphogenesis, and photosynthesis. Some key DEPs involved in storage substance decomposition, carbohydrate and energy metabolism were first activated during Phase I, and large amounts of nutrition and energy were needed following imbibition. Phase II included abundant metabolism and cellular activity, the appearance of the radicle and germ, abundant expression of numerous proteins involved in amino acid metabolism, protein synthesis, signal transduction, and cellular activity, which were beneficial for physiological processes and morphogenesis. Due to time constraints, only RuBisCO, which is related to the absence of O_2_ or photosynthesis deficiency, was detected during Phase III. In addition, due to moisture changes after imbibition, many proteins involved in plant defense emerged. Some key DEPs involved in storage substance degradation and plant defense mechanisms were found to be phosphorylated during seed germination, which may improve storage substance degradation and defense against environmental changes. Our results provide new evidence from the proteome and phosphorylation modification levels regarding the molecular mechanisms underlying plant seed germination.

## Author contributions

KD, SZ, and ZC carried out all experiments and data analysis. HC performed the preparation of protein, identification of phosphorylated proteins and bioinformatics analyses. PG made modifications for the manuscript. YY conceived the study, planned experiments, and helped draft the manuscript. All authors read and approved the final manuscript.

### Conflict of interest statement

The authors declare that the research was conducted in the absence of any commercial or financial relationships that could be construed as a potential conflict of interest.

## Abbreviations

2D-DIGE: two-dimensional differential gel electrophoresis
BAM: beta-amylase
DEP: differentially expressed protein
HAI: hours after imbibition
LC-MS/MS: liquid chromatography coupled to tandem mass spectrometry
MALDI-TOF/TOF-MS: matrix-assisted laser desorption/ionization time-of-flight tandem mass spectrometry
NCBI: National Center for Biotechnology Information
P^3^DB: Plant Protein Phosphorylation Data Base
PPI: protein-protein interaction
RuBisco: ribulose-1,5-bisphosphate carboxylase/oxygenase
SEM: scanning electronic microscope
SS: sucrose synthase.
