# Supplementary material for: Proteomic Analysis Reveals Key Proteins and Phosphoproteins upon Seed Germination of Wheat (Triticum aestivum L.)
Source: Front Plant Sci. 2015 Nov 18;6:1017. doi: 10.3389/fpls.2015.01017 (PMC4649031; doi:10.3389/fpls.2015.01017)
Supplement: Supplementary file 1 [file Table1.PDF]

Supplemental Table S1. Details of 2D-DIGE experiments for protein expression analysis

Gel Number	Cy2(Internal Standard-Pool)	Cy3(Sample)	Cy5(Sample)
Gel 1	0 HAI-1 + 12 HAI-1 + 24 HAI-1 + 36 HAI-1 + 48 HAI -1 + 0 HAI-2 + 12 HAI-2 + 24 HAI-2 + 36 HAI-2 + 48 HAI-2 + 0 HAI-3 + 12 HAI-3 + 24 HAI-3 + 36 HAI-3 + 48 HAI-3 + 0 HAI-4 + 12 HAI-4 + 24 HAI-4 + 36 HAI-4 + 48 HAI - 4	0 HAI-1*	12 HAI-1
Gel 2		12 HAI-2	24 HAI-1
Gel 3		24 HAI-2	36 HAI-1
Gel 4		36 HAI-2	48 HAI-1
Gel 5		48 HAI-2	0 HAI-2
Gel 6		0 HAI-3	24 HAI-3
Gel 7		24 HAI-4	48 HAI-3
Gel 8		48 HAI-4	12 HAI-3
Gel 9		12 HAI-4	36 HAI-3
Gel 10		36 HAI-4	0 HAI-4

*0 HAI-1: 0 HAI, 0 hour after imbibition, 1, the first biological replicate, every sample has four replicates.
