# Supplementary material for: Proteomic Analysis Reveals Key Proteins and Phosphoproteins upon Seed Germination of Wheat (Triticum aestivum L.)
Source: Front Plant Sci. 2015 Nov 18;6:1017. doi: 10.3389/fpls.2015.01017 (PMC4649031; doi:10.3389/fpls.2015.01017)
Supplement: Supplementary file 2 [file Table2.PDF]

Supplemental Table S2. DEPs identified by MALDI-TOF MS and MALDI-TOF/TOF MS in wheat germinations at five stages

**Spot**[a)]	**Protein Name**	**Accession No.**[b)]	**Species**[c)]	**Molecular function**[d)]	**Cellular component**[e)]	**Biological process**[f)]	**Differently expression**[g)]	**Identify**[h)]
**Carbohydrate metabolism**								
**1. Alcoholic fermentation**								
68	Alcohol dehydrogenase ADH1A	gi|119388723	*T. aestivum*	Oxidase	Cytoplasm	Short-chain alcohol metabolism	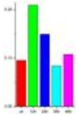	MS
256	Alcohol dehydrogenase ADH1A	gi|119388723	*T. aestivum*	Oxidase	Cytoplasm	Short-chain alcohol metabolism	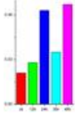	MS
**2. Glycolysis/Calvin cycle reactions**								
3	Glyceraldehyde-3-phosphate dehydrogenase, Cytosolic, GAPDH	gi|7579064	*T. aestivum*	Dehydrogenase	Cytosol	Energy production/Carbon skeleton synthesis	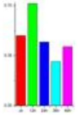	MSMS
11	Glyceraldehyde-3-phosphate dehydrogenase	gi|148508784	*T. aestivum*	Dehydrogenase	Cytosol/Chloroplast/ Nucleus	Energy production/Carbon skeleton synthesis/Gene expression/Posttranscriptional regulation	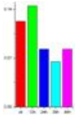	MSMS
221	Triosephosphat-isomerase	gi|11124572	*T. aestivum*	Allergen/Isomerase	Cytosol	Allergic reactions/Glycolysis pathway	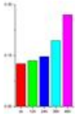	MSMS
224	Triosephosphat-isomerase	gi|11124572	*T. aestivum*	Allergen/Isomerase	Cytosol	Allergic reactions/Glycolysis pathway	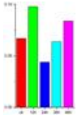	MSMS

Supplemental Table S2 continued

Spot[a)]	Protein Name	Accession No.[b)]	Species[c)]	Molecular function[d)]	Cellular component[e)]	Biological process[f)]	Differently expression[g)]	Identify[h)]
**3. Carbon skeletons**								
46	Phosphoenolpyruvate carboxylase	gi|56785833	*E. lanceolatus*	Carboxylase	Cytosol	Response to abiotic stresses affecting water status/Providing carbon skeletons	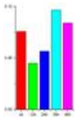	MS
**4. Starch synthesis/Degradation**								
6	Sucrose synthase type I	gi|3393067	*T. aestivum*	Synthetase	Cytoplasm	Sucrose metabolism/Energy metabolism/Controlling the mobilization of sucrose	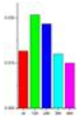	MSMS
228	Sucrose synthase type I	gi|3393067	*T. aestivum*	Synthetase	Cytoplasm	Sucrose metabolism/Energy metabolism/Controlling the mobilization of sucrose	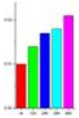	MSMS
40	Sucrose synthase type 2	gi|3393044	*T. aestivum*	Synthetase	Cytoplasm	Sucrose metabolism/Energy metabolism/Controlling the mobilization of sucrose	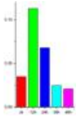	MSMS
138	Sucrose synthase type 2	gi|3393044	*T. aestivum*	Synthetase	Cytoplasm	Sucrose metabolism/Energy metabolism/Controlling the mobilization of sucrose	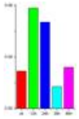	MS
27	UDP-glucoronosyl/UDP-glucosyl transferase protein	gi|171674071	*T. aestivum*	Glycosyltransferase	Endoplasmic reticulum membrane	Sustaining the carbon flux towards the fungus/Stress responses	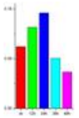	MS

Supplemental Table S2 continued

**Spot**[a)]	**Protein Name**	**Accession No.**[b)]	**Species**[c)]	**Molecular function**[d)]	**Cellular component**[e)]	**Biological process**[f)]	**Differently expression**[g)]	**Identify**[h)]
**4. Starch synthesis/Degradation**								
18	Beta amylase	gi|32400764	*T. aestivum*	Exoamylase/Hydrolase	Germplasm	Malting and brewing to convert starch into fermentable sugars	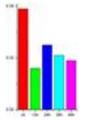	MSMS
82	Beta amylase	gi|32400764	*T. aestivum*	Exoamylase/Hydrolase	Germplasm	Malting and brewing to convert starch into fermentable sugars	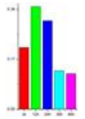	MSMS
88	Beta amylase	gi|32400764	*T. aestivum*	Exoamylase/Hydrolase	Germplasm	Malting and brewing to convert starch into fermentable sugars	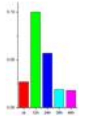	MS
91	Beta amylase	gi|32400764	*T. aestivum*	Exoamylase/Hydrolase	Germplasm	Malting and brewing to convert starch into fermentable sugars	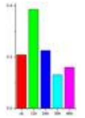	MS
98	Beta amylase	gi|32400764	*T. aestivum*	Exoamylase/Hydrolase	Germplasm	Malting and brewing to convert starch into fermentable sugars	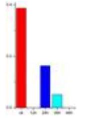	MS
102	Beta amylase	gi|32400764	*T. aestivum*	Exoamylase/Hydrolase	Germplasm	Malting and brewing to convert starch into fermentable sugars	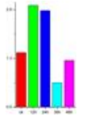	MS
141	Beta amylase	gi|32400764	*T. aestivum*	Exoamylase/Hydrolase	Germplasm	Malting and brewing to convert starch into fermentable sugars	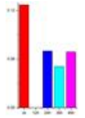	MSMS

Supplemental Table S2 continued

Spot[a)]	Protein Name	Accession No.[b)]	Species[c)]	Molecular function[d)]	Cellular component[e)]	Biological process[f)]	Differently expression[g)]	Identify [h)]
**4. Starch synthesis/Degradation**								
182	Beta amylase	gi|32400764	*T. aestivum*	Exoamylase/Hydrolase	Germplasm	Malting and brewing to convert starch into fermentable sugars	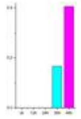	MSMS
209	Beta amylase	gi|32400764	*T. aestivum*	Exoamylase/Hydrolase	Germplasm	Malting and brewing to convert starch into fermentable sugars	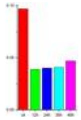	MSMS
276	Beta amylase	gi|32400764	*T. aestivum*	Exoamylase/Hydrolase	Germplasm	Malting and brewing to convert starch into fermentable sugars	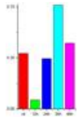	MS
297	Beta amylase	gi|32400764	*T. aestivum*	Exoamylase/Hydrolase	Germplasm	Malting and brewing to convert starch into fermentable sugars	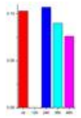	MS
328	Beta amylase	gi|32400764	*T. aestivum*	Exoamylase/Hydrolase	Germplasm	Malting and brewing to convert starch into fermentable sugars	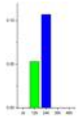	MSMS
330	Beta amylase	gi|32400764	*T. aestivum*	Exoamylase/Hydrolase	Germplasm	Malting and brewing to convert starch into fermentable sugars	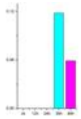	MS
47	Beta amylase	gi|1771782	*T. aestivum*	Exoamylase/Hydrolase	Germplasm	Malting and brewing to convert starch into fermentable sugars	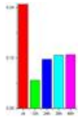	MSMS

Supplemental Table S2 continued

Spot[a)]	Protein Name	Accession No.[b)]	Species[c)]	Molecular function[d)]	Cellular component[e)]	Biological process[f)]	Differently expression[g)]	Identify[h)]
**4. Starch synthesis/Degradation**								
301	Beta amylase	gi|1771782	*T. aestivum*	Exoamylase/Hydrolase	Germplasm	Malting and brewing to convert starch into fermentable sugars	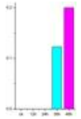	MSMS
86	Beta-amylase	gi|3334120	*T. aestivum*	Exoamylase/Hydrolase	Germplasm	Malting and brewing to convert starch into fermentable sugars	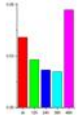	MSMS
233	ADP-glucose-pyrophosphorylase large subunit	gi|2583072	*T. aestivum*	Regulatory enzyme/ Catalyzing enzyme	Plastid/Cytosol	Starch biosynthesis	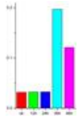	MS
269	Plastid ADP-glucose pyrophosphorylase small subunit	gi|224021585	*T. aestivum*	Regulatory enzyme/ Catalyzing enzyme	Plastid	Starch biosynthesis	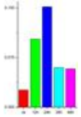	MSMS
**Storage proteins**								
**1. Globulin**								
4	Globulin 3	gi|215398470	*T. aestivum*	Storage protein/Allergen	Endosperm	Allergic reaction	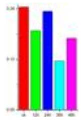	MS
5	Globulin 3	gi|215398470	*T. aestivum*	Storage protein/Allergen	Endosperm	Allergic reaction	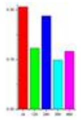	MSMS

Supplemental Table S2 continued

Spot[a)]	Protein Name	Accession No.[b)]	Species[c)]	Molecular function[d)]	Cellular component[e)]	Biological process[f)]	Differently expression[g)]	Identify[h)]
**1. Globulin**								
9	Globulin 3	gi|215398470	*T. aestivum*	Storage protein/Allergen	Endosperm	Allergic reaction	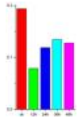	MSMS
19	Globulin 3	gi|215398470	*T. aestivum*	Storage protein/Allergen	Endosperm	Allergic reaction	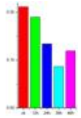	MS
25	Globulin 3	gi|215398470	*T. aestivum*	Storage protein/Allergen	Endosperm	Allergic reaction	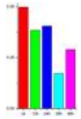	MS
26	Globulin 3	gi|215398470	*T. aestivum*	Storage protein/Allergen	Endosperm	Allergic reaction	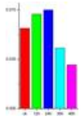	MSMS
29	Globulin 3	gi|215398470	*T. aestivum*	Storage protein/Allergen	Endosperm	Allergic reaction	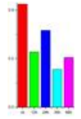	MS
36	Globulin 3	gi|215398470	*T. aestivum*	Storage protein/Allergen	Endosperm	Allergic reaction	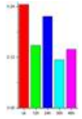	MSMS
41	Globulin 3	gi|215398470	*T. aestivum*	Storage protein/Allergen	Endosperm	Allergic reaction	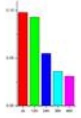	MSMS

Supplemental Table S2 continued

**Spot**[a)]	**Protein Name**	**Accession No.**[b)]	**Species**[c)]	**Molecular function**[d)]	**Cellular component**[e)]	**Biological process**[f)]	**Differently expression**[g)]	**Identify** [h)]
**1. Globulin**								
45	Globulin 3	gi|215398470	*T. aestivum*	Storage protein/Allergen	Endosperm	Allergic reaction	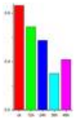	MS
59	Globulin 3	gi|215398470	*T. aestivum*	Storage protein/Allergen	Endosperm	Allergic reaction	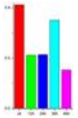	MSMS
64	Globulin 3	gi|215398470	*T. aestivum*	Storage protein/Allergen	Endosperm	Allergic reaction	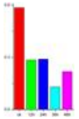	MS
66	Globulin 3	gi|215398470	*T. aestivum*	Storage protein/Allergen	Endosperm	Allergic reaction	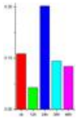	MSMS
72	Globulin 3	gi|215398470	*T. aestivum*	Storage protein/Allergen	Endosperm	Allergic reaction	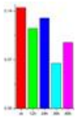	MSMS
77	Globulin 3	gi|215398470	*T. aestivum*	Storage protein/Allergen	Endosperm	Allergic reaction	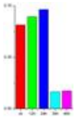	MSMS
78	Globulin 3	gi|215398470	*T. aestivum*	Storage protein/Allergen	Endosperm	Allergic reaction	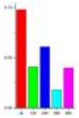	MS

Supplemental Table S2 continued

Spot[a)]	Protein Name	Accession No.[b)]	Species[c)]	Molecular function[d)]	Cellular component[e)]	Biological process[f)]	Differently expression[g)]	Identify[h)]
**1. Globulin**								
111	Globulin 3	gi|215398470	*T. aestivum*	Storage protein/Allergen	Endosperm	Allergic reaction	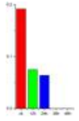	MS
112	Globulin 3	gi|215398470	*T. aestivum*	Storage protein/Allergen	Endosperm	Allergic reaction	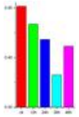	MS
113	Globulin 3	gi|215398470	*T. aestivum*	Storage protein/Allergen	Endosperm	Allergic reaction	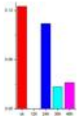	MS
155	Globulin 3	gi|215398470	*T. aestivum*	Storage protein/Allergen	Endosperm	Allergic reaction	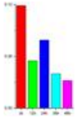	MS
166	Globulin 3	gi|215398470	*T. aestivum*	Storage protein/Allergen	Endosperm	Allergic reaction	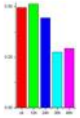	MS
167	Globulin 3	gi|215398470	*T. aestivum*	Storage protein/Allergen	Endosperm	Allergic reaction	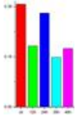	MS
170	Globulin 3	gi|215398470	*T. aestivum*	Storage protein/Allergen	Endosperm	Allergic reaction	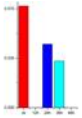	MS

Supplemental Table S2 continued

Spot[a)]	Protein Name	Accession No.[b)]	Species[c)]	Molecular function[d)]	Cellular component[e)]	Biological process[f)]	Differently expression[g)]	Identify[h)]
**1. Globulin**								
183	Globulin 3	gi|215398470	*T. aestivum*	Storage protein/Allergen	Endosperm	Allergic reaction	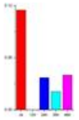	MS
184	Globulin 3	gi|215398470	*T. aestivum*	Storage protein/Allergen	Endosperm	Allergic reaction	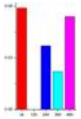	MS
185	Globulin 3	gi|215398470	*T. aestivum*	Storage protein/Allergen	Endosperm	Allergic reaction	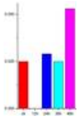	MS
240	Globulin 3	gi|215398470	*T. aestivum*	Storage protein/Allergen	Endosperm	Allergic reaction	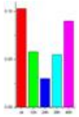	MSMS
251	Globulin 3	gi|215398470	*T. aestivum*	Storage protein/Allergen	Endosperm	Allergic reaction	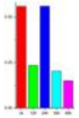	MS
272	Globulin 3	gi|215398470	*T. aestivum*	Storage protein/Allergen	Endosperm	Allergic reaction	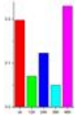	MS
295	Globulin 3	gi|215398470	*T. aestivum*	Storage protein/Allergen	Endosperm	Allergic reaction	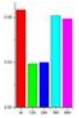	MSMS

Supplemental Table S2 continued

Spot[a)]	Protein Name	Accession No.[b)]	Species[c)]	Molecular function[d)]	Cellular component[e)]	Biological process[f)]	Differently expression[g)]	Identify[h)]
**1. Globulin**								
296	Globulin 3	gi|215398470	*T. aestivum*	Storage protein/Allergen	Endosperm	Allergic reaction	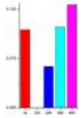	MS
314	Globulin 3	gi|215398470	*T. aestivum*	Storage protein/Allergen	Endosperm	Allergic reaction	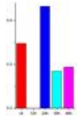	MSMS
323	Globulin 3	gi|215398470	*T. aestivum*	Storage protein/Allergen	Endosperm	Allergic reaction	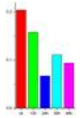	MSMS
336	Globulin 3	gi|215398470	*T. aestivum*	Storage protein/Allergen	Endosperm	Allergic reaction	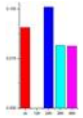	MS
337	Globulin 3	gi|215398470	*T. aestivum*	Storage protein/Allergen	Endosperm	Allergic reaction	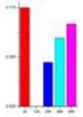	MS
343	Globulin 3	gi|215398470	*T. aestivum*	Storage protein/Allergen	Endosperm	Allergic reaction	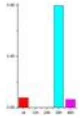	MSMS
52	Globulin 3B	gi|215398472	*T. aestivum*	Storage protein/Allergen	Endosperm	Allergic reaction	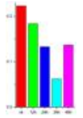	MSMS

Supplemental Table S2 continued

Spot[a)]	Protein Name	Accession No.[b)]	Species[c)]	Molecular function[d)]	Cellular component[e)]	Biological process[f)]	Differently expression[g)]	Identify[h)]
**1. Globulin**								
54	Globulin 3B	gi|215398472	*T. aestivum*	Storage protein/Allergen	Endosperm	Allergic reaction	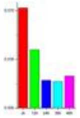	MSMS
55	Globulin 3B	gi|215398472	*T. aestivum*	Storage protein/Allergen	Endosperm	Allergic reaction	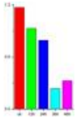	MSMS
315	Globulin 3B	gi|215398472	*T. aestivum*	Storage protein/Allergen	Endosperm	Allergic reaction	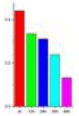	MSMS
43	Triticin	gi|171027826	*T. aestivum*	Globulin storage protein/Inhibitor	Endosperm	Ribosome inactivation/ Inhibitors of translation	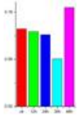	MSMS
121	Triticin	gi|171027826	*T. aestivum*	Globulin storage protein/Inhibitor	Endosperm	Ribosome inactivation/ Inhibitors of translation	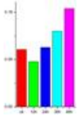	MSMS
217	Triticin precursor	gi|7548844	*T. aestivum*	Globulin storage protein/Inhibitor	Endosperm	Response to the abiotic or biotic stress.	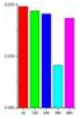	MSMS
234	Triticin precursor	gi|7548844	*T. aestivum*	Globulin storage protein/Inhibitor	Endosperm	Response to the abiotic or biotic stress	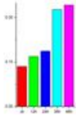	MSMS

Supplemental Table S2 continued

Spot[a)]	Protein Name	Accession No.[b)]	Species[c)]	Molecular function[d)]	Cellular component[e)]	Biological process[f)]	Differently expression[g)]	Identify[h)]
**1. Globulin**								
307	Triticin precursor	gi|7548844	*T. aestivum*	Globulin storage protein/Inhibitor	Endosperm	Response to the abiotic or biotic stress	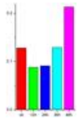	MSMS
319	19 kDa globulin	gi|32400820	*T. aestivum*	Storage protein	Endosperm	Relation to metal stress/ Activation of storage proteins	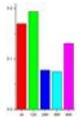	MSMS
**2. Gliadin**								
212	Alpha gliadin	gi|154268814	*T. aestivum*	Storage protein/Epitope	Endoplasmic reticulum/ Endosperm	T cell stimulatory epitopes/ Responsible for the properties of wheat products	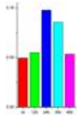	MSMS
**3. Glutenin**								
110	High-molecular-weight glutenin subunit y10	gi|164457873	*T. aestivum*	Storage protein	Endoplasmic reticulum/ Endosperm	Gluten formation and structure/Correlation with bread-making quality	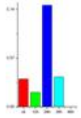	MSMS
163	HMW glutenin	gi|29569237	*T. aestivum*	Storage protein	Endoplasmic reticulum/ Endosperm	Gluten formation and structure/Correlation with bread-making quality	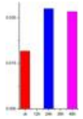	MSMS
165	HMW glutenin subunit 1By9	gi|22090	*T. aestivum*	Storage protein	Endoplasmic reticulum/ Endosperm	Gluten formation and structure/Correlation with bread-making quality	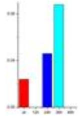	MSMS

Supplemental Table S2 continued

Spot[a)]	Protein Name	Accession No.[b)]	Species[c)]	Molecular function[d)]	Cellular component[e)]	Biological process[f)]	Differently expression[g)]	Identify[h)]
**3. Glutenin**								
266	X-type HMW glutenin	gi|110341791	*T. aestivum*	Storage protein	Endoplasmic reticulum/ Endosperm	Gluten formation and structure/Correlation with bread-making quality	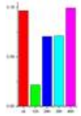	MSMS
278	X-type HMW glutenin	gi|110341791	*T. aestivum*	Storage protein	Endoplasmic reticulum/ Endosperm	Gluten formation and structure/Correlation with bread-making quality	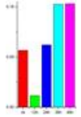	MSMS
283	HMW glutenin subunit	gi|45533903	*T. aestivum*	Storage protein	Endoplasmic reticulum/ Endosperm	Gluten formation and structure/Correlation with bread-making quality	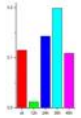	MSMS
286	High-molecular-weight glutenin subunit	gi|33414316	*T. aestivum*	Storage protein	Endoplasmic reticulum/ Endosperm	Gluten formation and structure/Correlation with bread-making quality	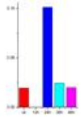	MSMS
294	Glutenin high molecular weight subunit	gi|24474920	*T. aestivum*	Storage protein	Endoplasmic reticulum/ Endosperm	Gluten formation and structure/Correlation with bread-making quality	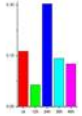	MSMS
122	Low molecular weight glutenin	gi|56480748	*T. aestivum*	Storage protein	Endoplasmic reticulum/ Endosperm	Gluten formation and structure/Correlation with bread-making quality	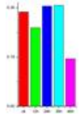	MSMS
219	LMW glutenin pGM107	gi|45477539	*T. aestivum*	Storage protein	Endoplasmic reticulum/ Endosperm	Gluten formation and structure/Correlation with bread-making quality	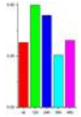	MSMS

Supplemental Table S2 continued

**Spot**[a)]	**Protein Name**	**Accession No.**[b)]	**Species**[c)]	**Molecular function**[d)]	**Cellular component**[e)]	**Biological process**[f)]	**Differently expression**[g)]	**Identify**[h)]
**3. Glutenin**								
274	Low molecular weight glutenin subunit	gi|26185819	*T. aestivum*	Storage protein	Endoplasmic reticulum/ Endosperm	Gluten formation and structure/Correlation with bread-making quality	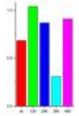	MSMS
339	S-type low molecular weight glutenin L4-292	gi|47607142	*T. aestivum*	Storage protein	Endoplasmic reticulum/ Endosperm	Gluten formation and structure/Correlation with bread-making quality	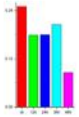	MSMS
**4. Other storage proteins**								
62	Avenin-like protein	gi|145321072	*T. aestivum*	Storage protein	Endosperm	Prolamins	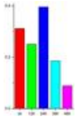	MSMS
327	Avenin-like protein	gi|145321072	*T. aestivum*	Storage protein	Endosperm	Prolamins	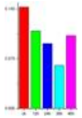	MSMS
**Energy metabolism**								
261	ATP synthase beta subunit	gi|525291	*T. aestivum*	Signalling molecules /Synthetase	Chloroplast/ Mitochondrial inner membrane/Thylakoid outer membrane	Nucleotide and protein binding/Synthesis and transformation of ATP	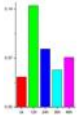	MS
277	Atp1	gi|81176509	*T. aestivum*	Signalling molecules	Mitochondria	Nucleotide and protein binding	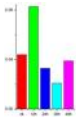	MS

Supplemental Table S2 continued

**Spot**[a)]	**Protein Name**	**Accession No.**[b)]	**Species**[c)]	**Molecular function**[d)]	**Cellular component**[e)]	**Biological process**[f)]	**Differently expression**[g)]	**Identify**[h)]
**Nitrogen metabolism**								
23	Aspartate aminotransferase	gi|164471780	*T. aestivum*	Transferase	Cytoplasm	Amino acid and nucleotide synthesis/Transferring nitrogen	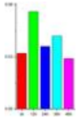	MS
229	Aspartate aminotransferase	gi|164471780	*T. aestivum*	Transferase	Cytoplasm	Amino acid and nucleotide synthesis/Transferring nitrogen	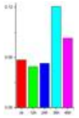	MSMS
**Cell metabolism**								
**1. Cell division**								
106	Cdc2-2D	gi|86439702	*T. aestivum*	Substrate	Embryo	Mitosis	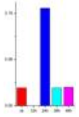	MS
218	Translationally-controlled tumor protein homolog (TCTP)	gi|75246527	*T. aestivum*	Regulatory protein/ Activator	Cytoplasm	Modulator of GTPase activity/ Cell division/Abiotic stresses/ Transformation/Egg cell fertilization/Pollen tube growth/ Auxin homeostasis	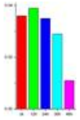	MS
293	Asynapsis 1	gi|148970484	*T. aestivum*	Regulator	Nucleus	Meiosis I	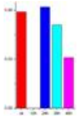	MS

Supplemental Table S2 continued

Spot[a)]	Protein Name	Accession No.[b)]	Species[c)]	Molecular function[d)]	Cellular component[e)]	Biological process[f)]	Differently expression[g)]	Identify[h)]
**2. Cell wall synthesis**								
10	Grain softness protein-1A	gi|60652210	*T. aestivum*	A marker protein for grain hardness	Endosperm	Controlling kernel hardness/ Cell wall structure	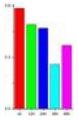	MS
116	Grain softness protein-1A	gi|60652210	*T. aestivum*	A marker protein for grain hardness	Endosperm	Controlling kernel hardness/ Cell wall structure	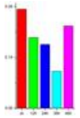	MS
2	Reversibly glycosylated polypeptide	gi|4158232	*T. aestivum*	The components	Cell wall/Golgi apparatus/Membrane/ Cytoplasm	Plant cell wall synthesis and regulation /Polysaccharide biosynthesis/Intercellular substance transportation and information transfer/Pollen development	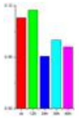	MS
211	Amylogenin	gi|4158230	*T. aestivum*	Glycoprotein	Cytoplasm	Synthesis of cell wall polysaccharides	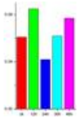	MS
**Transcription/Translation/Transposition**								
24	Transposase	gi|18419557	*T. aestivum*	Transposase	Nucleus	Transferring the transposon from the donor position into the new acceptor site	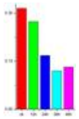	MS

Supplemental Table S2 continued

Spot[a]	Protein Name	Accession No.[b]	Species[c]	Molecular function[d]	Cellular component[e]	Biological process[f]	Differently expression[g]	Identify[h]
**Transcription/Translation/Transposition**								
15	Eukaryotic initiation factor 4B	gi|6739515	*T. aestivum*	Translational initiation factors	Ribosome	Apoptosis/Inhibition of protein synthetic activity/Interacting with the cap/Stimulating the RNA helicase activity of eIF4A/ Mediating mRNA binding to ribosomes	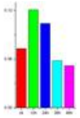	MS
117	Elongation factor 1-alpha	gi|219734387	*T. aestivum*	Elongation factors	Endosperm/Germ	Mediating the binding of aminoacyl-tRNA to the ribosome/mRNA localization/ Phosphatidylinositol 4-kinase regulation/Apoptosis/ Determination of longevity/ Major cytoskeleton-associated protein/Binding and bundling both microtubules or microfilaments/Co-localised with actin/depolymerisation of microfilaments/Colocalising with microfilaments/changes in intracellular pH	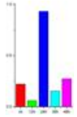	MSMS

Supplemental Table S2 continued

Spot[a)]	Protein Name	Accession No.[b)]	Species[c)]	Molecular function[d)]	Cellular component[e)]	Biological process[f)]	Differently expression[g)]	Identify[h)]
**Transcription/Translation/Transposition**								
92	Tritin	gi|391929	*T. aestivum*	Inhibitor	Endosperm	Ribosome inactivation	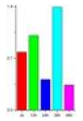	MS
118	Eukaryotic initiation factor 4A	gi|1170509	-----	Translational initiation factors	Ribosome	ATP-stimulated RNA-binding/RNA-dependent ATPase activity/RNA helicase activity/mRNA translation	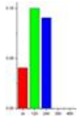	MSMS
284	60S ribosomal protein L36 (RPL36C)	gi|15241824	*A. thaliana*	Ribosomal protein	Cytosolic large ribosomal subunit/ ribosome/plasma membrane	Structural constituent of ribosome/Translation	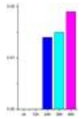	MS
**Photosynthesis**								
32	RuBisCO small subunit	gi|132107	*T. aestivum*	Carboxylase/Oxygenase	Chloroplast stroma	CO_2 fixation/Production of 2-phosphoglycolate	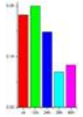	MS
81	RuBisCO small subunit	gi|132107	*T. aestivum*	Carboxylase/Oxygenase	Chloroplast stroma	CO_2 fixation/Production of 2-phosphoglycolate	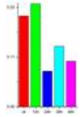	MS
109	RuBisCO small subunit	gi|132107	*T. aestivum*	Carboxylase/Oxygenase	Chloroplast stroma	CO_2 fixation/Production of 2-phosphoglycolate	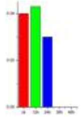	MSMS

Supplemental Table S2 continued

**Spot**[a)]	**Protein Name**	**Accession No.**[b)]	**Species**[c)]	**Molecular function**[d)]	**Cellular component**[e)]	**Biological process**[f)]	**Differently expression**[g)]	**Identify**[h)]
**Photosynthesis**								
201	RuBisCO small subunit	gi|132107	*T. aestivum*	Carboxylase/Oxygenase	Chloroplast stroma	CO_2 fixation/Production of 2-phosphoglycolate	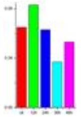	MS
238	RuBisCO small subunit	gi|132107	*T. aestivum*	Carboxylase/Oxygenase	Chloroplast stroma	CO_2 fixation/Production of 2-phosphoglycolate	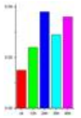	MS
257	RuBisCO small subunit	gi|132107	*T. aestivum*	Carboxylase/Oxygenase	Chloroplast stroma	CO_2 fixation/Production of 2-phosphoglycolate	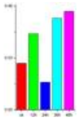	MS
264	RuBisCO small subunit	gi|132107	*T. aestivum*	Carboxylase/Oxygenase	Chloroplast stroma	CO_2 fixation/Production of 2-phosphoglycolate	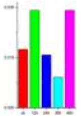	MS
252	Ribulose-1,5-bisphosphate carboxylase /oxygenase small subunit	gi|4038719	*T. aestivum*	Carboxylase/Oxygenase	Chloroplast stroma	CO_2 fixation/Production of 2-phosphoglycolate	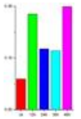	MS
255	Ribulose-bisphosphate carboxylase small chain precursor	gi|82619	*T. aestivum*	Carboxylase/Oxygenase	Chloroplast stroma	CO_2 fixation/Production of 2-phosphoglycolate	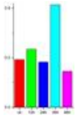	MS
322	Ribulose-1,5-bisphosphate carboxylase/oxygenase small subunit	gi|4038721	*T. aestivum*	Carboxylase/Oxygenase	Chloroplast stroma	CO_2 fixation/Production of 2-phosphoglycolate	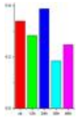	MS

Supplemental Table S2 continued

**Spot**[a)]	**Protein Name**	**Accession No.**[b)]	**Species**[c)]	**Molecular function**[d)]	**Cellular component**[e)]	**Biological process**[f)]	**Differently expression**[g)]	**Identify**[h)]
**Photosynthesis**								
289	Ribulose-1,5-bisphosphate carboxylase/oxygenase	gi|170771	*T. aestivum*	Carboxylase/Oxygenase	Chloroplast stroma	CO_2 fixation/Production of 2-phosphoglycolate	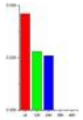	MS
309	Ribulose-1,5-bisphosphate carboxylase/oxygenase	gi|170771	*T. aestivum*	Carboxylase/Oxygenase	Chloroplast stroma	CO_2 fixation/Production of 2-phosphoglycolate	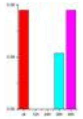	MS
**Signal transduction**								
14	ETTIN-like auxin response factor (ARF3)	gi|36939190	*T. aestivum*	Transcription factors	Embryo	Signal transduction/Pistil development/Determination of abaxial cell fate/Regulated gene expression mediated by auxin/ Impaired flower development	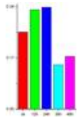	MS
28	ETTIN-like auxin response factor	gi|36939190	*T. aestivum*	Transcription factors	Embryo	Signal transduction/Pistil development/Determination of abaxial cell fate/Regulated gene expression mediated by auxin/ Impaired flower development	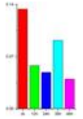	MS
79	Unnamed protein product	gi|18616497	*T. aestivum*	Regulatory protein NPR1	Cytoplasm	Resistance/Transcription factors/Regulating DNA binding activity.	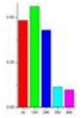	MS

Supplemental Table S2 continued

**Spot**[a)]	**Protein Name**	**Accession No.**[b)]	**Species**[c)]	**Molecular function**[d)]	**Cellular component**[e)]	**Biological process**[f)]	**Differently expression**[g)]	**Identify**[h)]
**Signal transduction**								
179	Small Ras-related GTP-binding protein	gi|16903082	*T. aestivum*	Membrane trafficking regulators	Membrane	DNA damage repair/toleration/ Nuclear translocation/Signal transduction/Control ciliary membrane biogenesis/Cellular processes	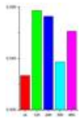	MSMS
**Stress/Defense/Detoxification**								
1	Peroxidase 1	gi|22001285	*T. aestivum*	Isoenzyme/ Bifunctional enzyme	Intracellular/Cell wall or the surrounding medium/Vacuole	Defense against abiotic or biotic stresses/Signaling/Cell wall hardening/cell elongation/Restrict cell growth /Auxin catabolism/Biosynthesis of secondary metabolites/ Oxidative metabolism	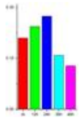	MS
34	Peroxidase 1	gi|22001285	*T. aestivum*	Isoenzyme/ Bifunctional enzyme	Intracellular/Cell wall or the surrounding medium/Vacuole	Defense against abiotic or biotic stresses/Signaling/Cell wall hardening/cell elongation/Restrict cell growth /Auxin catabolism/Biosynthesis of secondary metabolites/ Oxidative metabolism	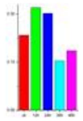	MS

Supplemental Table S2 continued

**Spot**[a)]	**Protein Name**	**Accession No.**[b)]	**Species**[c)]	**Molecular function**[d)]	**Cellular component**[e)]	**Biological process**[f)]	**Differently expression**[g)]	**Identify**[h)]
**Stress/Defense/Detoxification**								
279	Peroxidase 1	gi|22001285	*T. aestivum*	Isoenzyme/Bifunctional enzyme	Intracellular/Cell wall or the surrounding medium/Vacuole	Defense against abiotic or biotic stresses/Signaling/Cell wall hardening/cell elongation/Restrict cell growth /Auxin catabolism/Biosynthesis of secondary metabolites/ Oxidative metabolism	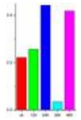	MS
12	LEA 1 protein	gi|25989705	*T. aestivum*	Late embryogenesis abundant protein	Cytoplasm/Nucleus/ Nucleolus/Mitochondria /Chloroplast/Vacuole/ Endoplasmic reticulum/ Perixosome/Plasma membrane	The acquisition of desiccation, salt, cold, and osmotic stresses	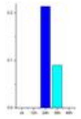	MSMS
22	Class II chitinase	gi|62465514	*T. aestivum*	Pathogen induced protein	Intermediate layer/ Aleurone layer	Inhibiting fungal growth/ Signaling/Releasing elicitor molecules	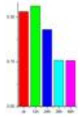	MSMS
135	Class II chitinase	gi|62465514	*T. aestivum*	Pathogen induced protein	Intermediate layer/ Aleurone layer	Inhibiting fungal growth/ Signaling/Releasing elicitor molecules	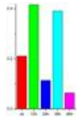	MSMS

Supplemental Table S2 continued

**Spot**[a)]	**Protein Name**	**Accession No.**[b)]	**Species**[c)]	**Molecular function**[d)]	**Cellular component**[e)]	**Biological process**[f)]	**Differently expression**[g)]	**Identify**[h)]
**Stress/Defense/Detoxification**								
136	Class II chitinase	gi|62465514	*T. aestivum*	Pathogen induced protein	Intermediate layer/ Aleurone layer	Inhibiting fungal growth/ Signaling/Releasing elicitor molecules	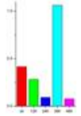	MSMS
231	Class II chitinase	gi|62465514	*T. aestivum*	Pathogen induced protein	Intermediate layer/ Aleurone layer	Inhibiting fungal growth/ Signaling/Releasing elicitor molecules	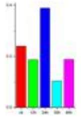	MSMS
267	Class II chitinase	gi|62465514	*T. aestivum*	Pathogen induced protein	Intermediate layer/ Aleurone layer	Inhibiting fungal growth/ Signaling/Releasing elicitor molecules	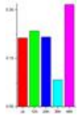	MSMS
270	Class II chitinase	gi|62465514	*T. aestivum*	Pathogen induced protein	Intermediate layer/ Aleurone layer	Inhibiting fungal growth/ Signaling/Releasing elicitor molecules	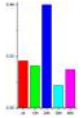	MSMS
37	Putative RNA-directed RNA polymerase	gi|133627	---	RNA polymerase	Intracellular	Posttranscriptional gene silencing	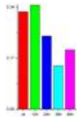	MS
39	Serpin	gi|1885346	*T. aestivum*	Allergens/A feeding deterrent/Inhibitor/Storage protein	Endosperm	Allergic reaction/Plant defense and/or oxidative stress/Storage protein	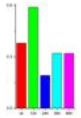	MSMS
51	Serpin	gi|1885346	*T. aestivum*	Allergens/A feeding deterrent/Inhibitor/Storage protein	Endosperm	Allergic reaction/Plant defense and/or oxidative stress/Storage protein	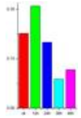	MSMS

Supplemental Table S2 continued

Spot[a)]	Protein Name	Accession No.[b)]	Species[c)]	Molecular function[d)]	Cellular component[e)]	Biological process[f)]	Differently expression[g)]	Identify[h)]
**Stress/Defense/Detoxification**								
232	Serpin	gi|1885346	*T. aestivum*	Allergens/A feeding deterrent/Inhibitor/Storage protein	Endosperm	Allergic reaction/Plant defense and/or oxidative stress/Storage protein	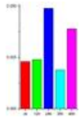	MSMS
73	Serpin 1	gi|224589266	*T. aestivum*	Allergens/A feeding deterrent/Inhibitor/Storage protein/Anti-elastase	Endosperm	Allergic reaction/Plant defense and/or oxidative stress/Storage protein	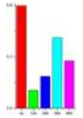	MS
254	Serpin 1	gi|224589266	*T. aestivum*	Allergens/A feeding deterrent/Inhibitor/Storage protein/Anti-elastase	Endosperm	Allergic reaction/Plant defense and/or oxidative stress/Storage protein	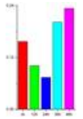	MS
306	Serpin 1	gi|224589266	*T. aestivum*	Allergens/A feeding deterrent/Inhibitor/Storage protein/Anti-elastase	Endosperm	Allergic reaction/Plant defense and/or oxidative stress/Storage protein	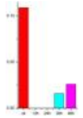	MS
308	Serpin 1	gi|224589266	*T. aestivum*	Allergens/A feeding deterrent/Inhibitor/Storage protein/Anti-elastase	Endosperm	Allergic reaction/Plant defense and/or oxidative stress/Storage protein	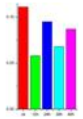	MS
76	Serpin-Z1A	gi|75282265	*T. aestivum*	Allergens/A feeding deterrent/Inhibitor/Storage protein	Endosperm	Allergic reaction/Plant defense and/or oxidative stress/Storage protein	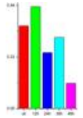	MS
119	Serpin-Z2B	gi|75279909	*T. aestivum*	Allergens/A feeding deterrent/Inhibitor/Storage protein	Endosperm	Allergic reaction/Plant defense and/or oxidative stress/Storage protein	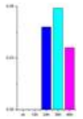	MSMS

Supplemental Table S2 continued

**Spot**[a)]	**Protein Name**	**Accession No.**[b)]	**Species**[c)]	**Molecular function**[d)]	**Cellular component**[e)]	**Biological process**[f)]	**Differently expression**[g)]	**Identify**[h)]
**Stress/Defense/Detoxification**								
120	Serpin-Z2B	gi|75279909	*T. aestivum*	Allergens/A feeding deterrent/Inhibitor/Storage protein	Endosperm	Allergic reaction/Plant defense and/or oxidative stress/Storage protein	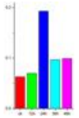	MSMS
213	Serpin-Z1C	gi|75313848	*T. aestivum*	Allergens/A feeding deterrent/Inhibitor/Storage protein	Endosperm	Allergic reaction/Plant defense and/or oxidative stress/Storage protein	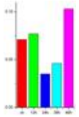	MSMS
61	Thioredoxin peroxidase	gi|75324900	*T. aestivum*	Peroxidase	Embryo	Cell detoxification/Regulating proteins	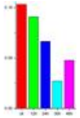	MS
215	Thioredoxin peroxidase	gi|75324900	*T. aestivum*	Peroxidase	Embryo	Cell detoxification/Regulating proteins	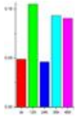	MS
63	Heat shock protein 16.8	gi|445135	*T. aestivum*	Molecular chaperone	Cytoplasm	To cope with cold stress, UV-B treatment and wounding treatment/Photosynthesis	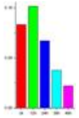	MSMS
205	Heat shock protein 101	gi|4558484	*T. aestivum*	Molecular chaperone	Endosperm/Embryo	Conferring thermotolerance in plants and yeast	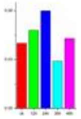	MS
207	Heat shock protein 101	gi|4558484	*T. aestivum*	Molecular chaperone	Endosperm/Embryo	Conferring thermotolerance in plants and yeast	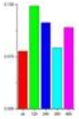	MS

Supplemental Table S2 continued

Spot[a]	Protein Name	Accession No.[b]	Species[c]	Molecular function[d]	Cellular component[e]	Biological process[f]	Differently expression[g]	Identify[h]
**Stress/Defense/Detoxification**								
204	Heat shock protein 90	gi|110270498	*T. aestivum*	Conserved molecular chaperone	Cytoplasm	Cell signaling/Signal transduction/Protein degradation/Protein folding and refolding/Immune response	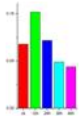	MS
230	Heat shock protein HSP26	gi|4028567	*T. aestivum*	Molecular chaperone	Chloroplast	Protecting the chloroplast from oxidative stress and heat stress	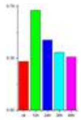	MS
332	Cold shock domain protein 3	gi|42391858	*T. aestivum*	RNA chaperone	Endoplasmic reticulum	Transcription anti-terminators/ Destabilizing RNA secondary structures /Exhibiting nucleic acid binding /Freezing tolerance	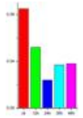	MSMS
235	Alpha amylase inhibitor	gi|225042	*T. aestivum*	Inhibitor	Endosperm	Against the attack of pests and herbivores	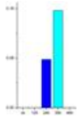	MS
271	Alpha amylase inhibitor	gi|225042	*T. aestivum*	Inhibitor	Endosperm	Against the attack of pests and herbivores	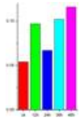	MS
236	Chain A, Crystal Structure Of Family 11 Xylanase In Complex With Inhibitor (Xip-I)	gi|51247633	*T. aestivum*	Inhibitors	Endosperm	Protecting the grain from pathogen attack	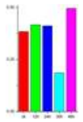	MS

Supplemental Table S2 continued

Spot[a)]	Protein Name	Accession No.[b)]	Species[c)]	Molecular function[d)]	Cellular component[e)]	Biological process[f)]	Differently expression[g)]	Identify[h)]
**Stress/Defense/Detoxification**								
243	Sequence 3 from patent US 6903246	gi|67622999	*T. aestivum*	Reductase	Cytoplasm	Modulating ascorbic acid levels /Ascorbate-glutathione recycling reaction	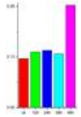	MS
248	5a2 protein	gi|66840998	*T. aestivum*	Amino acid fragment	Endosperm	Response to stress	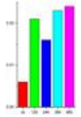	MS
263	WRKY45 transcription factor	gi|126508732	*T. aestivum*	Transcriptional regulators	Nucleus	Controlling the transcription of defense genes/Salicylic acid signaling pathway	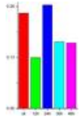	MS
280	Resistance protein	gi|3320431	*T. aestivum*	Recognition of pathogen effectors	Cytoplasm	Innate immune system	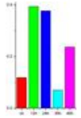	MS
325	Similar to puroindoline a	gi|48093961	*T. aestivum*	Cystine-rich protein/A marker protein for grain hardness	Endosperm	Controlling grain texture/ Binding to lipids	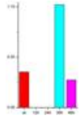	MSMS
**Unknown**								
101	Unnamed protein product	gi|219766623	*T. aestivum*	-	-	-	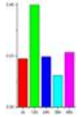	MS

Supplemental Table S2 continued

Spot[a)]	Protein Name	Accession No.[b)]	Species[c)]	Molecular function[d)]	Cellular component[e)]	Biological process[f)]	Differently expression[g)]	Identify[h)]
**Unknown**								
275	Unnamed protein product	gi|219766623	*T. aestivum*	-	-	-	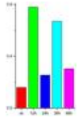	MS
210	Unknown protein	gi|10801369	*A. thaliana*	-	-	-	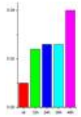	MS
305	Unnamed protein product	gi|219765726	*T. aestivum*	-	-	-	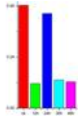	MSMS

a) Spot: numbers correspond to protein spot on gels shown in Figure 2.

b) Accession No.: number of predicted protein in NCBInr.

c) Species: the species of predicted protein in NCBInr.

d) Molecular function: the function of differentially expressed proteins in molecular level.

e) Cellular component: the location of differentially expressed proteins in wheat seed.

f) Biological process: the predicted biological processes which differentially expressed proteins involved in.

g) Differently expression: the expression type of predicted protein in wheat germination at five stages. CK, 12h, 24h, 36h, 48h.

h) Identify: identify ways to confirm the kind of differentially expressed proteins.
