# Supplementary material for: Proteomic Analysis Reveals Key Proteins and Phosphoproteins upon Seed Germination of Wheat (Triticum aestivum L.)
Source: Front Plant Sci. 2015 Nov 18;6:1017. doi: 10.3389/fpls.2015.01017 (PMC4649031; doi:10.3389/fpls.2015.01017)
Supplement: Supplementary file 4 [file Table4.PDF]

Supplemental Table S4. %Vol of all 2-fold DEPs

Spot[a)]	Protein name	0 HAI	12 HAI	24 HAI	36 HAI	48 HAI
1	Peroxidase 1	0.210	0.244	0.274	0.159	0.128
2	Reversibly glycosylated polypeptide	0.106	0.115	0.061	0.080	0.072
3	glyceraldehyde-3-phosphate dehydrogenase, Cytosolic, GAPDH	0.490	0.714	0.445	0.308	0.413
4	Globulin 3	0.244	0.188	0.234	0.116	0.170
5	Globulin 3	0.371	0.221	0.338	0.178	0.210
6	Sucrose synthase type I	0.019	0.031	0.028	0.018	0.015
9	Globulin 3	0.194	0.079	0.119	0.135	0.128
10	Grain softness protein-1A	0.586	0.491	0.470	0.259	0.370
11	Glyceraldehyde-3-phosphate dehydrogenase	0.123	0.145	0.083	0.065	0.083
12	LEA 1 protein	-	-	0.214	0.090	-
14	ETTIN-like auxin response factor	0.256	0.330	0.338	0.147	0.175
15	Eukaryotic initiation factor 4B	0.073	0.121	0.104	0.058	0.052
18	Beta amylase	0.039	0.016	0.025	0.021	0.019
19	Globulin 3	0.317	0.285	0.201	0.130	0.179
22	Class II chitinase	0.318	0.337	0.258	0.155	0.154
23	Aspartate aminotransferase	0.032	0.056	0.036	0.042	0.029
24	Transposase	0.315	0.274	0.168	0.120	0.132
25	Globulin 3	0.558	0.431	0.454	0.194	0.325
26	Globulin 3	0.057	0.067	0.070	0.043	0.031
27	UDP-glucoronosyl/UDP-glucosyl transferase protein	0.112	0.147	0.173	0.091	0.066
28	ETTIN-like auxin response factor	0.134	0.058	0.049	0.092	0.040
29	Globulin 3	0.425	0.228	0.317	0.156	0.205
32	RuBisCO small subunit	0.255	0.279	0.208	0.098	0.117
34	Peroxidase 1	0.234	0.320	0.302	0.154	0.186
36	Globulin 3	0.245	0.148	0.217	0.114	0.139
37	Putative RNA-directed RNA polymerase	0.325	0.347	0.244	0.145	0.198
39	Serpin	0.380	0.590	0.192	0.321	0.320
40	Sucrose synthase type 2	0.035	0.113	0.068	0.025	0.021
41	Globulin 3	0.098	0.093	0.055	0.036	0.031
43	Triticin	0.148	0.143	0.137	0.091	0.189
45	Globulin 3	0.864	0.688	0.576	0.302	0.417
46	Phosphoenolpyruvate carboxylase	0.091	0.054	0.068	0.116	0.101
47	Beta amylase	0.248	0.067	0.117	0.127	0.128
51	Serpin	0.227	0.310	0.200	0.088	0.117
52	Globulin 3B	0.223	0.184	0.133	0.062	0.137
54	Globulin 3B	0.072	0.042	0.020	0.019	0.023
55	Globulin 3B	1.172	0.931	0.794	0.238	0.327
59	Globulin 3	0.412	0.212	0.214	0.351	0.153

Supplemental Table S4 continued

Spot[a)]	Protein name	0 HAI	12 HAI	24 HAI	36 HAI	48 HAI
61	Thioredoxin peroxidase	0.186	0.164	0.120	0.049	0.086
62	Avenin-like protein	0.312	0.251	0.396	0.186	0.089
63	Heat shock protein 16.8	0.150	0.183	0.121	0.068	0.040
64	Globulin 3	0.585	0.285	0.290	0.130	0.218
66	Globulin 3	0.163	0.064	0.303	0.142	0.126
68	Alcohol dehydrogenase ADH1A	0.124	0.272	0.194	0.109	0.140
72	Globulin 3	0.145	0.115	0.130	0.065	0.095
73	Serpin 1	0.598	0.105	0.186	0.411	0.276
76	Serpin-Z1A	0.353	0.435	0.239	0.304	0.108
77	Globulin 3	0.570	0.626	0.675	0.115	0.121
78	Globulin 3	0.098	0.041	0.061	0.018	0.040
79	Unnamed protein product	0.483	0.561	0.430	0.113	0.097
81	RuBisCO small subunit	0.201	0.228	0.079	0.134	0.101
82	Beta amylase	0.210	0.349	0.301	0.131	0.121
86	Beta-amylase	0.041	0.028	0.022	0.021	0.057
88	Beta amylase	0.027	0.100	0.057	0.019	0.018
91	Beta amylase	0.209	0.386	0.226	0.131	0.160
92	Tritin	0.786	1.009	0.416	1.395	0.341
98	Beta amylase	0.387	-	0.163	0.051	-
101	Unnamed protein product	0.218	0.460	0.227	0.143	0.246
102	Beta amylase	1.454	2.716	2.578	0.648	1.247
106	Cdc2-2D	0.031	-	0.169	0.031	0.032
109	RuBisCO small subunit	0.040	0.043	0.030	-	-
110	High-molecular-weight glutenin subunit y10	0.040	0.021	0.146	0.043	-
111	Globulin 3	0.192	0.075	0.064	-	-
112	Globulin 3	0.916	0.754	0.615	0.294	0.554
113	Globulin 3	0.125	-	0.104	0.027	0.032
116	Grain softness protein-1A	0.273	0.195	0.176	0.102	0.227
117	Elongation factor 1-alpha	0.221	0.061	0.933	0.154	0.274
118	Eukaryotic initiation factor 4A	0.065	0.160	0.145	-	-
119	Serpin-Z2B	-	-	0.048	0.059	0.036
120	Serpin-Z2B	0.063	0.070	0.193	0.097	0.099
121	Triticin	0.109	0.086	0.113	0.144	0.187
122	Low molecular weight glutenin	0.345	0.287	0.366	0.369	0.174
135	Class II chitinase	0.211	0.416	0.114	0.391	0.064
136	Class II chitinase	0.416	0.282	0.092	1.062	0.077
138	Sucrose synthase type 2	0.029	0.078	0.067	0.017	0.032
141	Beta amylase	0.127	-	0.070	0.051	0.069
155	Globulin 3	0.119	0.055	0.079	0.040	0.032
163	HMW glutenin	0.019	-	0.033	-	0.032

Supplemental Table S4 continued

Spot[a)]	Protein name	0 HAI	12 HAI	24 HAI	36 HAI	48 HAI
165	HMW glutenin subunit 1By9	0.024	-	0.046	0.088	-
166	Globulin 3	0.495	0.513	0.444	0.275	0.293
167	Globulin 3	0.369	0.219	0.337	0.178	0.210
170	Globulin 3	0.072	-	0.045	0.033	-
179	Small Ras-related GTP-binding protein	0.030	0.087	0.082	0.042	0.069
182	Beta amylase	-	-	-	0.167	0.406
183	Globulin 3	0.115	-	0.037	0.021	0.040
184	Globulin 3	0.059	-	0.037	0.022	0.054
185	Globulin 3	0.025	-	0.029	0.025	0.053
201	RuBisCO small subunit	0.065	0.083	0.063	0.037	0.053
204	Heat shock protein 90	0.122	0.183	0.129	0.088	0.079
205	Heat shock protein 101	0.040	0.048	0.060	0.029	0.043
207	Heat shock protein 101	0.083	0.148	0.124	0.088	0.117
209	Beta amylase	0.097	0.039	0.0401	0.0409	0.047
210	Unknown protein	0.010	0.024	0.026	0.026	0.040
211	Amylogenin	0.061	0.085	0.042	0.062	0.077
212	Alpha gliadin	0.089	0.099	0.176	0.154	0.096
213	Serpin-Z1C	0.071	0.077	0.035	0.046	0.103
215	Thioredoxin peroxidase	0.088	0.189	0.083	0.168	0.163
217	Triticin precursor	0.069	0.066	0.064	0.029	0.061
218	Translationally-controlled tumor protein homolog (TCTP)	0.036	0.039	0.035	0.029	0.011
219	LMW glutenin pGM107	0.570	0.900	0.809	0.460	0.589
221	Triosephosphat-isomerase	0.126	0.135	0.147	0.194	0.270
224	Triosephosphat-isomerase	0.067	0.098	0.044	0.064	0.084
228	Sucrose synthase type I	0.010	0.014	0.017	0.018	0.021
229	Aspartate aminotransferase	0.057	0.049	0.053	0.121	0.083
230	Heat shock protein HSP26	0.329	0.673	0.473	0.390	0.359
231	Class II chitinase	0.240	0.187	0.389	0.104	0.188
232	Serpin	0.023	0.024	0.049	0.019	0.039
233	ADP-glucose-pyrophosphorylase large subunit	0.0318	0.0323	0.0326	0.197	0.121
234	Triticin precursor	0.135	0.169	0.187	0.327	0.342
235	Alpha amylase inhibitor	-	-	0.078	0.157	-
236	Chain A, Crystal Structure Of Family 11 Xylanase In Complex With Inhibitor (Xip-I)	0.384	0.417	0.412	0.186	0.496
238	RuBisCO small subunit	0.015	0.024	0.038	0.029	0.036
240	Globulin 3	0.103	0.058	0.030	0.055	0.090
243	Sequence 3 from patent US 6903246	0.126	0.143	0.147	0.138	0.263
248	5a2 protein	0.006	0.021	0.016	0.023	0.024

Supplemental Table S4 continued

Spot[a)]	Protein name	0 HAI	12 HAI	24 HAI	36 HAI	48 HAI
251	Globulin 3	0.559	0.235	0.560	0.203	0.150
252	Ribulose-1,5-bisphosphate carboxylase /oxygenase small subunit	0.107	0.333	0.212	0.205	0.359
254	Serpin 1	0.157	0.101	0.074	0.202	0.234
255	Ribulose-bisphosphate carboxylase small chain precursor	0.193	0.235	0.183	0.414	0.146
256	Alcohol dehydrogenase ADH1A	0.021	0.028	0.063	0.035	0.067
257	RuBisCO small subunit	0.027	0.044	0.016	0.053	0.057
261	ATP synthase beta subunit	0.043	0.145	0.083	0.053	0.071
263	WRKY45 transcription factor	0.243	0.130	0.264	0.170	0.167
264	RuBisCO small subunit	0.021	0.035	0.019	0.011	0.035
266	X-type HMW glutenin	0.174	0.039	0.127	0.128	0.179
267	Class II chitinase	0.213	0.235	0.216	0.083	0.317
269	Plastid ADP-glucose pyrophosphorylase small subunit	0.026	0.103	0.152	0.060	0.058
270	Class II chitinase	0.201	0.180	0.440	0.096	0.164
271	Alpha amylase inhibitor	0.054	0.097	0.067	0.102	0.116
272	Globulin 3	0.198	0.070	0.123	0.050	0.230
274	Low molecular weight glutenin subunit	0.822	1.249	1.043	0.372	1.093
275	Unnamed protein product	0.160	0.780	0.255	0.671	0.304
276	Beta amylase	0.382	0.062	0.346	0.713	0.452
277	Atp1	0.044	0.083	0.033	0.021	0.039
278	X-type HMW glutenin	0.102	0.021	0.112	0.185	0.186
279	Peroxidase 1	0.222	0.257	0.443	0.035	0.419
280	Resistance protein	0.119	0.394	0.377	0.070	0.237
283	HMW glutenin subunit	0.115	0.012	0.143	0.199	0.109
284	60S ribosomal protein L36 (RPL36C)	-	-	0.014	0.015	0.019
286	High-molecular-weight glutenin subunit	0.031	-	0.162	0.039	0.032
289	Ribulose-1,5-bisphosphate carboxylase/oxygenase	0.046	0.028	0.026	-	-
293	Asynapsis 1	0.059	-	0.062	0.051	0.031
294	Glutenin high molecular weight subunit	0.163	0.064	0.303	0.142	0.126
295	Globulin 3	0.065	0.029	0.030	0.061	0.059
296	Globulin 3	0.119	-	0.063	0.123	0.157
297	Beta amylase	0.103	-	0.107	0.090	0.076
301	Beta amylase	-	-	-	0.123	0.200
305	Unnamed protein product	0.484	0.116	0.445	0.133	0.124
306	Serpin 1	0.109	-	-	0.016	0.026
307	Triticin precursor	0.128	0.088	0.091	0.129	0.213
308	Serpin 1	0.111	0.058	0.095	0.068	0.087

Supplemental Table S4 continued

Spot[a)]	Protein name	0 HAI	12 HAI	24 HAI	36 HAI	48 HAI
309	Ribulose-1,5-bisphosphate carboxylase /oxygenase	0.076	-	-	0.043	0.076
314	Globulin 3	0.296	-	0.465	0.169	0.188
315	Globulin 3B	0.442	0.336	0.312	0.240	0.133
319	19 kDa globulin	0.170	0.194	0.078	0.075	0.131
322	Ribulose-1,5-bisphosphate carboxylase/oxygenase small subunit	0.340	0.284	0.388	0.185	0.248
323	Globulin 3	0.204	0.158	0.067	0.111	0.094
325	Similar to puroindoline a	0.389	-	-	1.124	0.302
327	Avenin-like protein	0.153	0.117	0.098	0.065	0.110
328	Beta amylase	-	0.053	0.107	-	-
330	Beta amylase	-	-	-	0.118	0.059
332	Cold shock domain protein 3	0.085	0.052	0.024	0.037	0.038
336	Globulin 3	0.122	-	0.153	0.095	0.094
337	Globulin 3	0.170	-	0.076	0.118	0.142
339	S-type low molecular weight glutenin L4-292	0.250	0.179	0.180	0.205	0.086
343	Globulin 3	0.086	-	-	0.896	0.071

a) Spot: numbers correspond to protein spot on gels shown in Figure 2.
