# Supplementary material for: Proteomic Analysis Reveals Key Proteins and Phosphoproteins upon Seed Germination of Wheat (Triticum aestivum L.)
Source: Front Plant Sci. 2015 Nov 18;6:1017. doi: 10.3389/fpls.2015.01017 (PMC4649031; doi:10.3389/fpls.2015.01017)
Supplement: Supplementary file 5 [file Table5.PDF]

Supplemental Table S5. KOG annotation of DEPs from Jimai 20 in the process of seed germination.

Gi number	Protein Descriptions	KOG number	KOG Descriptions
gi|119388723|gb|ABL74258.1|	Alcohol dehydrogenase ADH1A [Triticum aestivum]	KOG0022	Alcohol dehydrogenase, class III
gi|7579064|gb|AAF64241.1|	Cytosolic glyceraldehyde-3-phosphate dehydrogenase GAPDH, partial [Triticum aestivum]	KOG0657	Glyceraldehyde 3-phosphate dehydrogenase
gi|148508784|gb|ABQ81648.1|	Glyceraldehyde-3-phosphate dehydrogenase [Triticum aestivum]	KOG0657	Glyceraldehyde 3-phosphate dehydrogenase
gi|11124572|emb|CAC14917.1|	Triosephosphat-isomerase [Triticum aestivum]	KOG1643	Triosephosphate isomerase
gi|3393067|emb|CAA04543.1|	Sucrose synthase type I [Triticum aestivum]	KOG0853	Glycosyltransferase
gi|3393044|emb|CAA03935.1|	Sucrose synthase type 2 [Triticum aestivum]	KOG0853	Glycosyltransferase
gi|171674071|gb|ACB47884.1|	UDP-glucoronosyl/UDP-glucosyl transferase protein [Triticum aestivum]	KOG1192	UDP-glucuronosyl and UDP-glucosyl transferase
gi|2583072|gb|AAB82604.1|	ADP-glucose-pyrophosphorylase large subunit, partial [Triticum aestivum]	KOG1322	GDP-mannose pyrophosphorylase/mannose-1-phosphate guanylyltransferase
gi|224021585|gb|ACN32622.1|	Plastid ADP-glucose pyrophosphorylase small subunit [Triticum aestivum]	KOG1322	GDP-mannose pyrophosphorylase/mannose-1-phosphate guanylyltransferase
gi|164471780|gb|ABY58643.1|	Aspartate aminotransferase, partial [Triticum aestivum]	KOG1411	Aspartate aminotransferase/Glutamic oxaloacetic transaminase AAT1/GOT2
gi|86439702|emb|CAJ19331.1|	Cdc2-2D [Triticum aestivum]	KOG0663	Protein kinase PITSLRE and related kinases
gi|75246527|sp|Q8LRM8.1|	TCTP_WHEAT RecName: Full=Translationally-controlled tumor protein homolog; Short=TCTP	KOG1727	Microtubule-binding protein (translationally controlled tumor protein)
gi|148970484|gb|ABR20128.1|	Asynapsis 1 [Triticum aestivum]	KOG4652	HORMA domain

Supplemental Table S5 continued

Gi number	Protein Descriptions	KOG number	KOG Descriptions
gi|525291|emb|CAA52636.1|	ATP synthase beta subunit [Triticum aestivum]	KOG1350	F0F1-type ATP synthase, beta subunit
gi|81176509|ref|YP_398393.1|	Atp1 (mitochondrion) [Triticum aestivum]	KOG1353	F0F1-type ATP synthase, alpha subunit
gi|16903082|gb|AAL30396.1|AF433653_1	Small Ras-related GTP-binding protein [Triticum aestivum]	KOG0096	GTPase Ran/TC4/GSP1 (nuclear protein transport pathway), small G protein superfamily [..]
gi|391929|dbj|BAA02948.1|	Tritin [Triticum aestivum]	KOG2402	Paf1/RNA polymerase II complex, RTF1 component (involved in regulation of TATA box-binding [..]
gi|219734387|emb|CAW34309.1|	Unnamed protein product [Triticum aestivum]	KOG0052	Translation elongation factor EF-1 alpha/Tu
gi|1170509|sp|P41378.1|	IF4A_WHEAT RecName: Full=Eukaryotic initiation factor 4A; Short=eIF-4A; AltName: Full=ATP-dependent RNA helicase eIF4A	KOG0327	Translation initiation factor 4F, helicase subunit (eIF-4A) and related helicases [..]
gi|15241824|ref|NP_195865.1|	60S ribosomal protein L36-3 [Arabidopsis thaliana]	KOG3452	60S ribosomal protein L36
gi|25989705|gb|AAN74637.1|	LEA1 protein [Triticum aestivum]	KOG4744	Uncharacterized conserved protein
gi|62465514|gb|AAX83262.1|	Class II chitinase [Triticum aestivum]	KOG4742	Predicted chitinase
gi|1885346|emb|CAA72274.1|	Serpin [Triticum aestivum]	KOG2392	Serpin
gi|224589266|gb|ACN59483.1|	Serpin 1 [Triticum aestivum]	KOG2392	Serpin
gi|75282265|sp|Q41593.1|	SPZ1A_WHEAT RecName: Full=Serpin-Z1A; AltName: Full=TriaeZ1a; AltName: Full=WSZ1a; Short=WSZ1; AltName: Full=WSZCI	KOG2392	Serpin
gi|75279909|sp|P93692.1|	SPZ2B_WHEAT RecName: Full=Serpin-Z2B; AltName: Full=TriaeZ2b; AltName: Full=WSZ2b; AltName: Full=WZS3	KOG2392	Serpin

Supplemental Table S5 continued

Gi number	Protein Descriptions	KOG number	KOG Descriptions
gi|75313848|sp|Q9ST58.1|	SPZ1C_WHEAT RecName: Full=Serpin-Z1C; AltName: Full=TriaeZ1c; AltName: Full=WSZ1c	KOG2392	Serpin
gi|75324900|sp|Q6W8Q2.1|	REHY_WHEAT RecName: Full=1-Cys peroxiredoxin PER1; AltName: Full=Rehydrin homolog; AltName: Full=Thioredoxin peroxidase	KOG0854	Alkyl hydroperoxide reductase, thiol specific antioxidant and related enzymes
gi|445135|prf||1908436A	Heat shock protein 16.8	KOG0710	Molecular chaperone (small heat-shock protein Hsp26/Hsp42)
gi|4558484|gb|AAD22629.1|AF097363_1	Heat shock protein 101 [Triticum aestivum]	KOG1051	Chaperone HSP104 and related ATP-dependent Clp proteases
gi|110270498|gb|ABG57075.1|	Heat shock protein 90 [Triticum aestivum]	KOG0019	Molecular chaperone (HSP90 family)
gi|4028567|gb|AAC96314.1|	Heat shock protein HSP26 [Triticum aestivum]	KOG0710	Molecular chaperone (small heat-shock protein Hsp26/Hsp42)
gi|42391858|dbj|BAD08701.1|	Cold shock domain protein 3 [Triticum aestivum]	KOG3070	Predicted RNA-binding protein containing PIN domain and invovled in translation or [..]
gi|18616497|emb|CAD22847.1|	Unnamed protein product [Triticum aestivum]	KOG0504	FOG: Ankyrin repeat
gi|51247633|pdb|1TE1|	A Chain A, Crystal Structure Of Family 11 Xylanase In Complex With Inhibitor (xip-i)	KOG4701	Chitinase
gi|67622999|gb|AAY78446.1|	Sequence 3 from patent US 6903246	KOG1422	Intracellular Cl- channel CLIC, contains GST domain
gi|3320431|gb|AAC26505.1|	Resistance protein, partial [Triticum aestivum]	KOG4658	Apoptotic ATPase
gi|48093961|gb|AAT40243.1|	Similar to puroindoline a, partial [Triticum aestivum]	KOG0065	Pleiotropic drug resistance proteins (PDR1-15), ABC superfamily
