# Supplementary material for: Proteomic Analysis Reveals Key Proteins and Phosphoproteins upon Seed Germination of Wheat (Triticum aestivum L.)
Source: Front Plant Sci. 2015 Nov 18;6:1017. doi: 10.3389/fpls.2015.01017 (PMC4649031; doi:10.3389/fpls.2015.01017)
Supplement: Supplementary file 8 [file Presentation1.PDF]

Supporting Information

Supplemental Figure S1. Proteome maps of wheat whole proteins in seed germination from five stages of Jimai 20. Protein samples (600 μg) extracted from five stages was separated on standard preparative 2-DE gels stained with CBB. Differentially expressed protein (DEP) spots were numbered on gel images.

Supplemental Figure S2. Images of phosphoproteins stained with Pro-Q Diamond. Protein spots were numbered on gel images, which were consistent with Figure 2.

Supplemental Figure S3. Prediction of phosphorylated modification sites on DEPs in germination. A: Prediction of phosphorylated modification sites on DEPs in NetPhos 2.0 Server; B: Prediction map of DEPs in NetPhos 2.0 Server.

Supplemental Figure S4. Sequence alignment of phosphorylated modification sites in P^3DB database on DEPs in germination. Blue box presented conserved phosphorylated modification sites in wheat and other varieties.

Supplemental Figure S5. Expression profiles of phosphorylated proteins. A: Images of phosphoproteins stained with Pro-Q Diamond; B: Expression profiles.

Supplemental Table S1. Details of 2D-DIGE experiments for protein expression analysis.

Supplemental Table S2. Differentially expressed proteins identified by MALDI-TOF MS and MALDI-TOF/TOF MS in wheat germinations at five stages

Supplemental Table S3. Peptide information of differentially expressed proteins identified by MALDI-TOF MS and MALDI-TOF/TOF MS

Supplemental Table S4. %Vol of all 2-fold DEPs

Supplemental Table S5. KOG annotation of DEPs from Jimai 20 in the process of seed germination.

Supplemental Table S6. Peptide information of phosphorylated proteins identified by MALDI-TOF/TOF MS

Supplemental Table S7. Phosphorylated proteins information searched in P^3DB database.
