# Supplementary figures and images for: Proteomic Analysis Reveals Key Proteins and Phosphoproteins upon Seed Germination of Wheat (Triticum aestivum L.)
Source: Front Plant Sci. 2015 Nov 18;6:1017. doi: 10.3389/fpls.2015.01017 (PMC4649031; doi:10.3389/fpls.2015.01017)

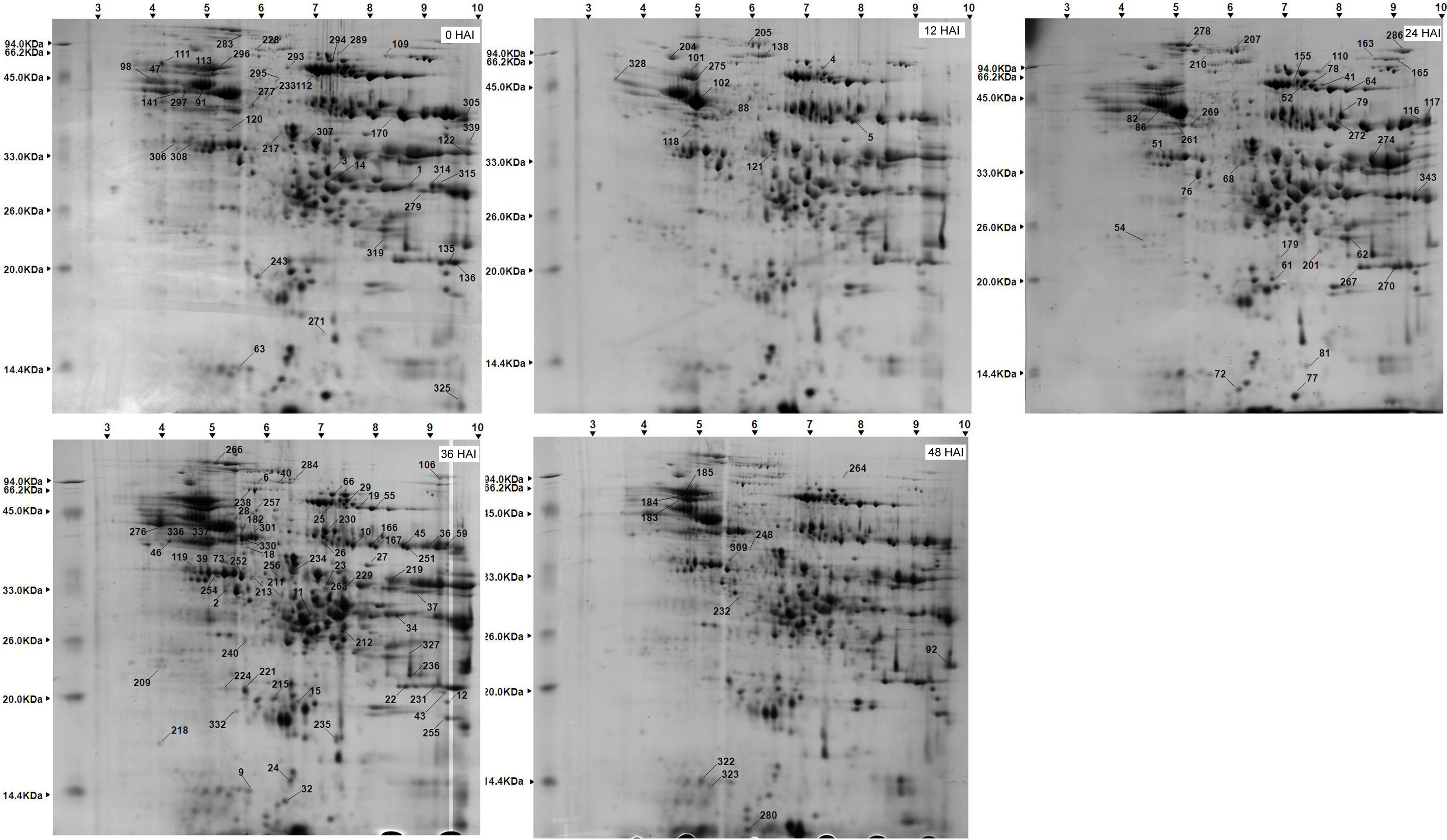

Supplement: Supplementary file 9 [file Image1.JPEG]

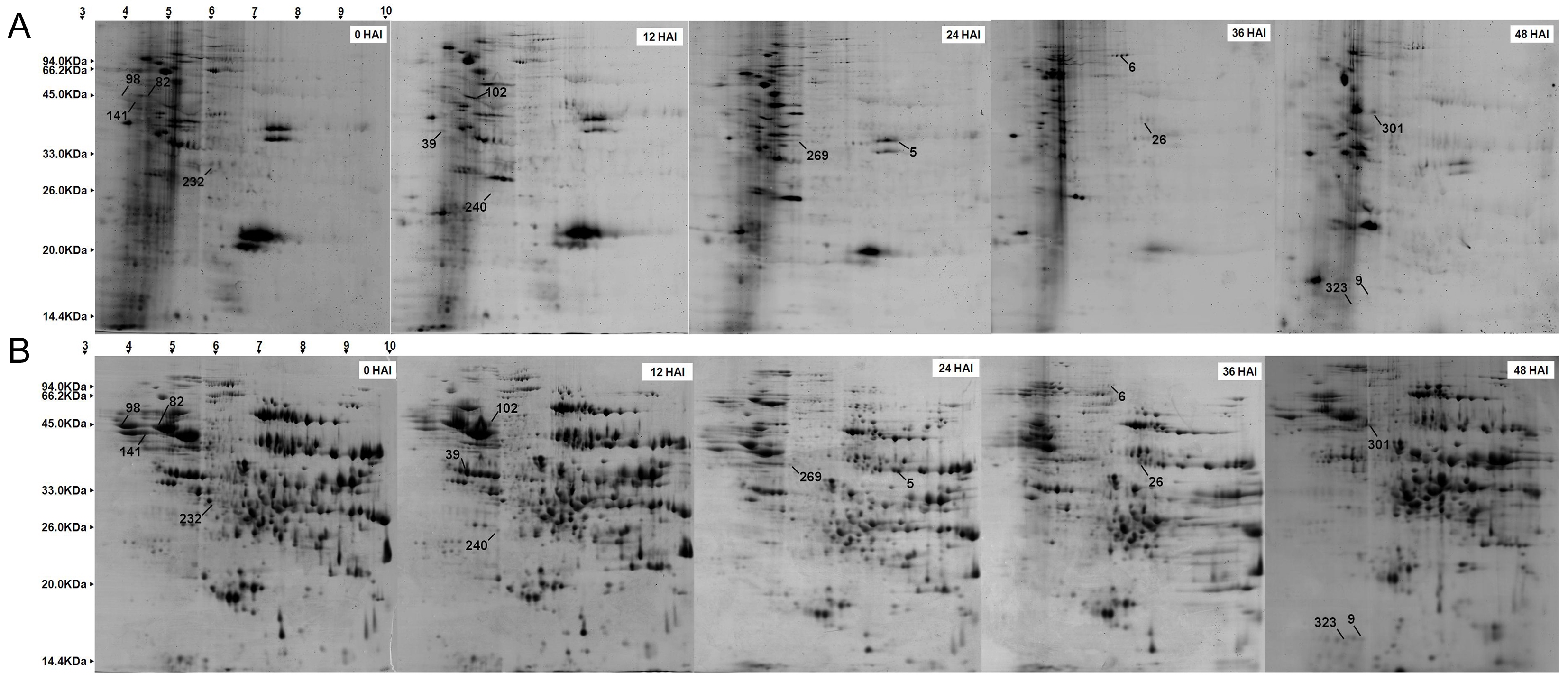

Supplement: Supplementary file 10 [file Image2.JPEG]

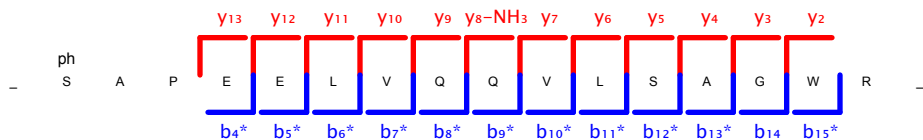

m/z (full)

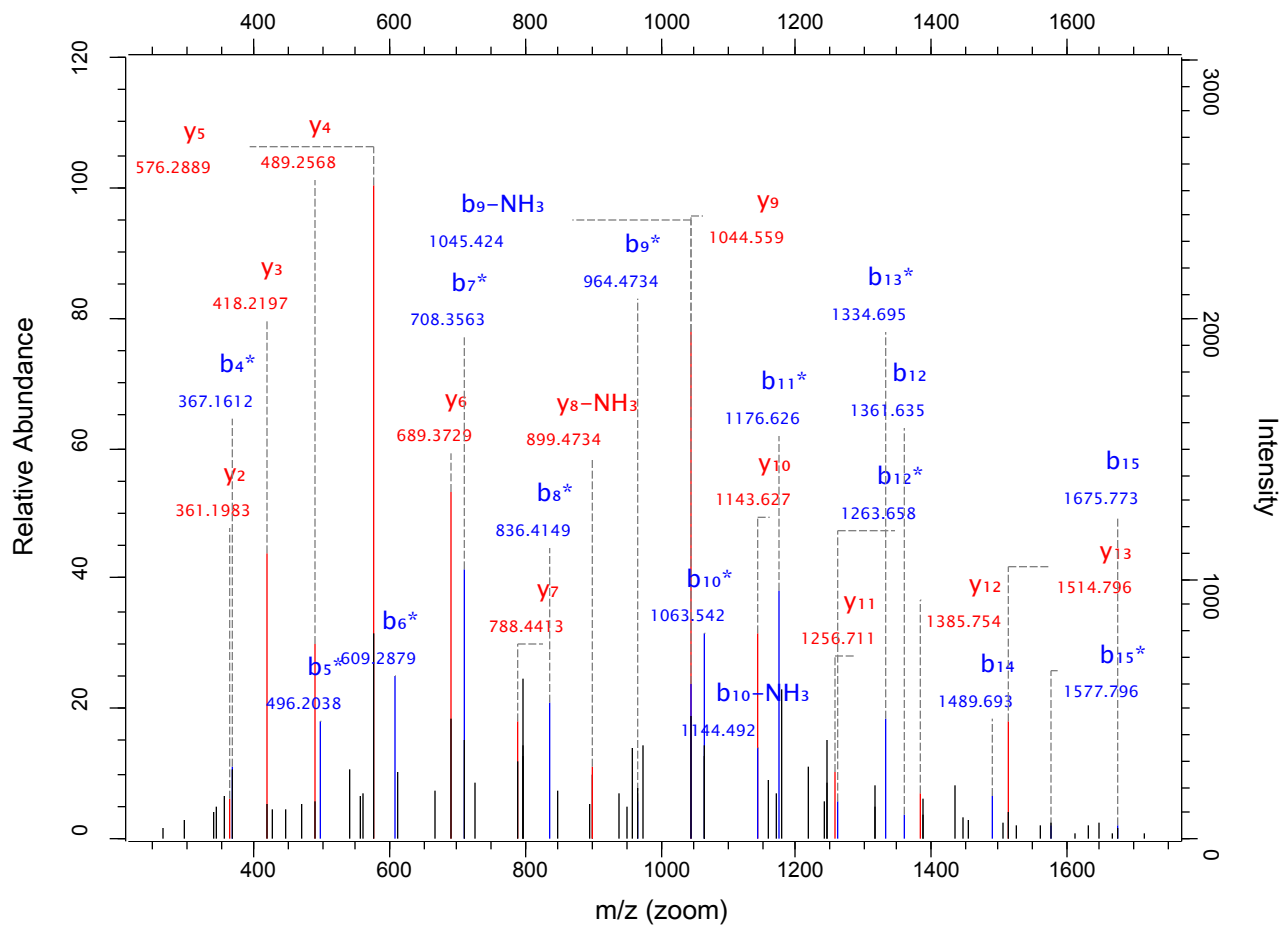

Supplement: Supplementary file 13 [file Image5.PDF]

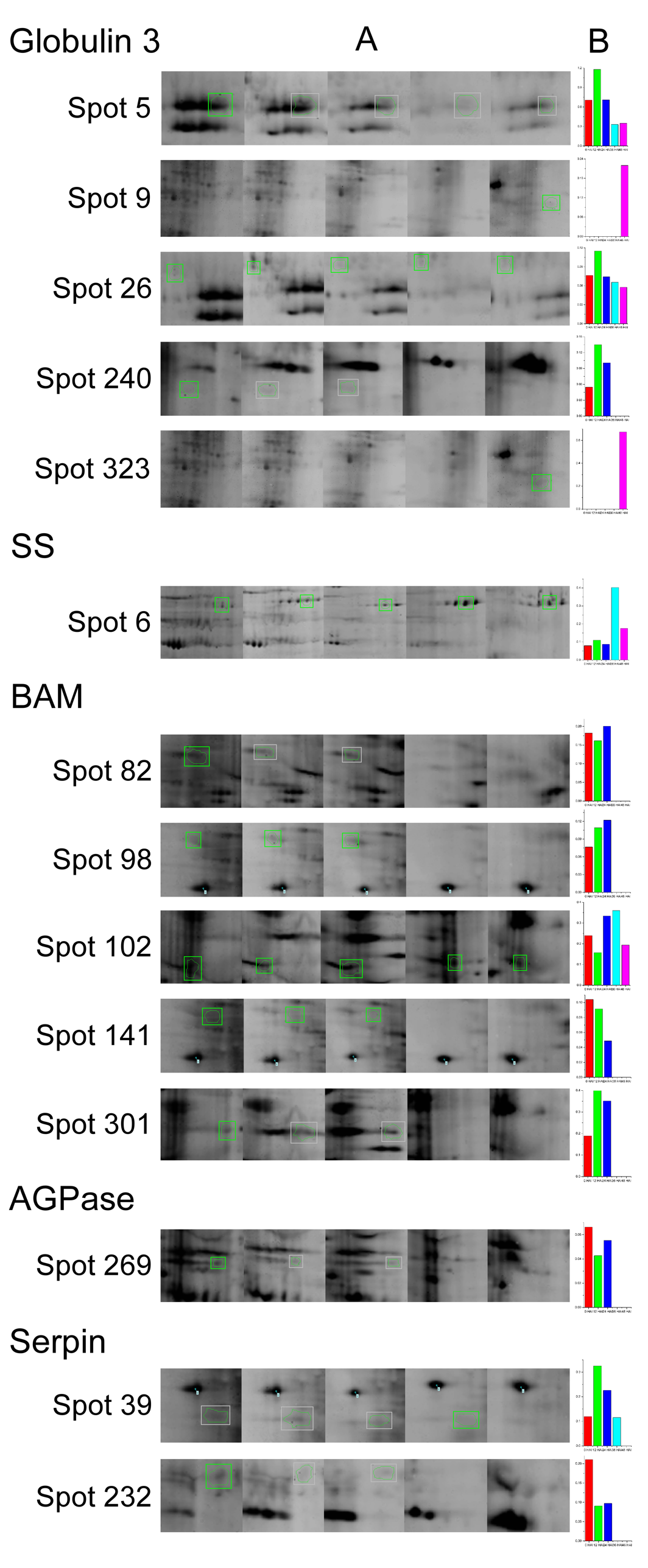

Supplement: Supplementary file 14 [file Image6.JPEG]
