# Supplementary material for: Proteomic Analysis Reveals Key Proteins and Phosphoproteins upon Seed Germination of Wheat (Triticum aestivum L.)
Source: Front Plant Sci. 2015 Nov 18;6:1017. doi: 10.3389/fpls.2015.01017 (PMC4649031; doi:10.3389/fpls.2015.01017)

Supplemental Figure S3. Prediction of phosphorylated modification sites on DEPs in germination. A: Prediction of phosphorylated modification sites on DEPs in NetPhos 2.0 Server; B: Prediction map of DEPs in NetPhos 2.0 Server.

Spot 5, 9, 26, 240 and 323 Globulin 3 *T. aestivum* gi|215398470 ACJ65514.1

A

```
588 Sequence
MATRARVTIPLLFLLGTSLLFAAAVSASHDEEEDRRGGRSLQQCVQRCQQDRPRYSHARCVQECRDDQQQHGRHEQEEQG     80
RGHGRHGEGEREEEQGRGRGRHGQGEREEEQGRGRGRRGEGERDEEHGDGRRPYVFGPRSFRRIIRSDHGFVKALRPFDE    160
VSRLLRGIRNYRVAIMEVNPRAFVVPGLTDADGVGYVAQGEGVLTVIENGEKRSYTVRQGDVIVAPAGSIMHLANTDGRR    240
KLVIAKILHTISVPGKFQYFSAKPLLASLSKRVLTAALKTSDERLGSLLGSRQGKEEEEKSISIVRASEEQLRELRRQAS    320
EGDQGHHWPLPPFRGDSRDTFNLLEQRPKIANRHGRLYEADARSFHALAQHDVRVAVANITPGSMTAPYLNTQSFKLAVV    400
LEGEGEVEIVCPHLGRDSERREQEHGKGRWRSEEEEDDRRQQRRRGSGSESEEEQDQQRYETVRARVSRGSAFVVPPGHP    480
VVEIASSRGSSNLQVVCFEINAERNERVWLAGRNNVIAKLDDPAQELTFGRPAREVQEVFRAKDQQDEGFVAGPEQQQEH    560
ERGDRRRGDRGRGDEAVEAFLRMATAAL                                                         640
.......T...................S...........................S........................     80
...........................................................S....................    160
...................................Y........T..........T........................    240
....................S..................TS.....S...............S....S...........S    320
...................T............................................................    400
.................S.............S..............S.S.S........Y.T.....S..S.........    480
.........S......................................................................    560
............................                                                         640

Phosphorylation sites predicted:      Ser: 17   Thr: 6   Tyr: 2
```

]

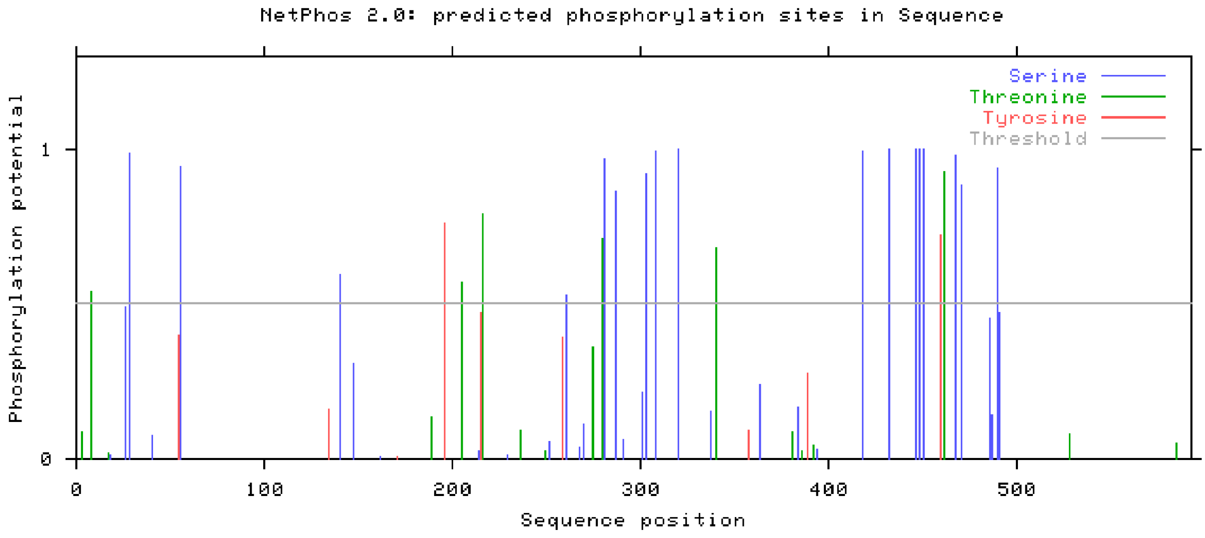

Spot 6 Sucrose synthase type I *T. aestivum* gi|3393067 CAA04543.1

A

```
808 Sequence
MAAKLTRLHSLRERLGATFSSHPNELIALFSRYVHQGKGMLQRHQLLAEFDALFESDKEKYAPFEDILRAAQEAIVLPPW      80
VALAIRPRPGVWDYIRVNVSELAVEELTVSEYLAFKEQLVDEHASSKFVLELDFEPFNASFPRPSMSNSIGKRVQFLNRH     160
LSSQLFQDKESLYPLLNFLKAHYYKGTTMMLNDRIQSLRGLQSALRKAEEYLVSIPEDTPSSEFNHRFQELGLEKGWGDT     240
AKRVHDTIHLLLDLLEAPDPASLEKFLGTIPMMFNVVILSPHGYFAQSNVLGYPDTGGQVVYILDQVRALENEMLLRIKQ     320
QGLDITPKILIVTRLLPDAVGTTCGQRLEKVIGTEHTDILRVPFRTDNGILRKWISRFDVWPYLETYTEDVANELMREMQ     400
TKPDFIIGNNSDGNLVATLLAHKLGVTQCTIAHALEKTKYPNSDIYLDKFDSQYHFSCQFTADLIAMNHTDFIITSTFQE     480
IAGSKDSVGQYESHIAFTLPDLYRVVHGIDVFDPKFNIVXPGADMTVYFPYTETDKRLTAFHSEIEELLYSDVENDEHKF     560
VLKDRNKPIIFSMARLDRVKNMTGLVEMYGKNAHLKGFGKLVIVAGDHGKESKDREEQAEFKRMYSLIEEYKLKGHIRWI     640
SAQMNRVRNGELYRYICDTKGAFVQPAFYEAFGLTVIEVHECGLPTIATCHGGPAEIIVNGVSGLHIDPYHSDKAADILV     720
NFFEKCSEDPSYWDKMSEGGLKRIYEKYTWKLYSERLMTLTGVYGFWKYVSNLERRETRRYLEMFYALKYRSLAAAVPLA     800
VDGESSDN                                                                             880
.........S.............................................S....Y...................      80
.............Y.............T.................S..................S...............     160
..........S.Y.....................................Y.......T.SS.................T     240
......T..............S..............................Y...........................     320
.....T...............T..........................................................     400
T.........S............................Y.....Y.......Y..........................     480
......S...Y..........................................T....T...S......YS.........     560
...................................................S.............S..............     640
.......................................................................S........     720
..........SY....S.....................T.........Y........T...........Y..........     800
........                                                                             880
```

Phosphorylation sites predicted: Ser: 17 Thr: 11 Tyr: 13

B

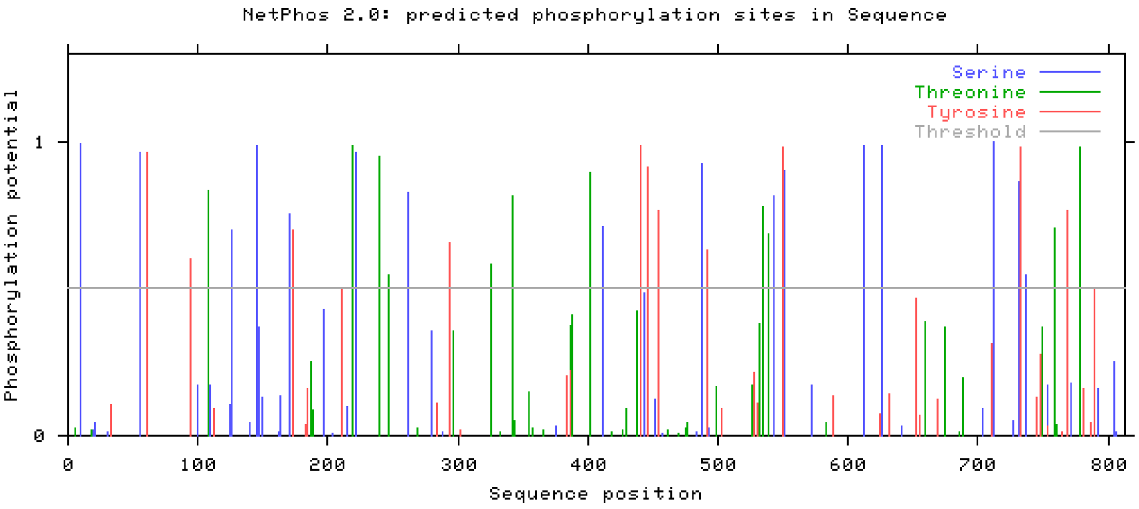

Spot 39 and 232 Serpin *T. aestivum* gi|1885346 CAA72274.1

A

```
398 Sequence
MATTLATDVRLSIAHQTRFAFRLASAISSNPESTVNNAAFSPVSLHVALSLITAGAGGATRNQLAATLGEGEVEGLHALA    80
EQVVQFVLADASNIGGPRVAFANGVFVDASLQLKPSFQELAVCKYKAEAQSVDFQTKAAEVTAQVNSWVEKVTTGLIKDI   160
LPAGSIDNTTRLVLGNALYFKGAWTDQFDPRATQSDDFYLLDGSSIQTPFMYSSEEQYISSSDGLKVLKLPYKQGGDKRQ   240
FSMYILLPEALSGLWSLAEKLSAEPEFLEQHIPRQKVALRQFKLPKFKISLGIEASDLLKGLGLLLPFGAEADLSEMVDS   320
PMAQNLYISSIFHKAFVEVNETGTEAAATTIAKVVLRQAPPPSVLDFIVDHPFLFLIREDTSGVVLFIGHVVNPLLSS     400
...........S....T...............ST......S.......................................    80
..................................................S...............S.............   160
....S...T.......................T.....Y........T....S....Y.S....................   240
.S...................S.........................................................S   320
.............................T............S.................TS................     400

Phosphorylation sites predicted:    Ser: 13   Thr: 7   Tyr: 2
```

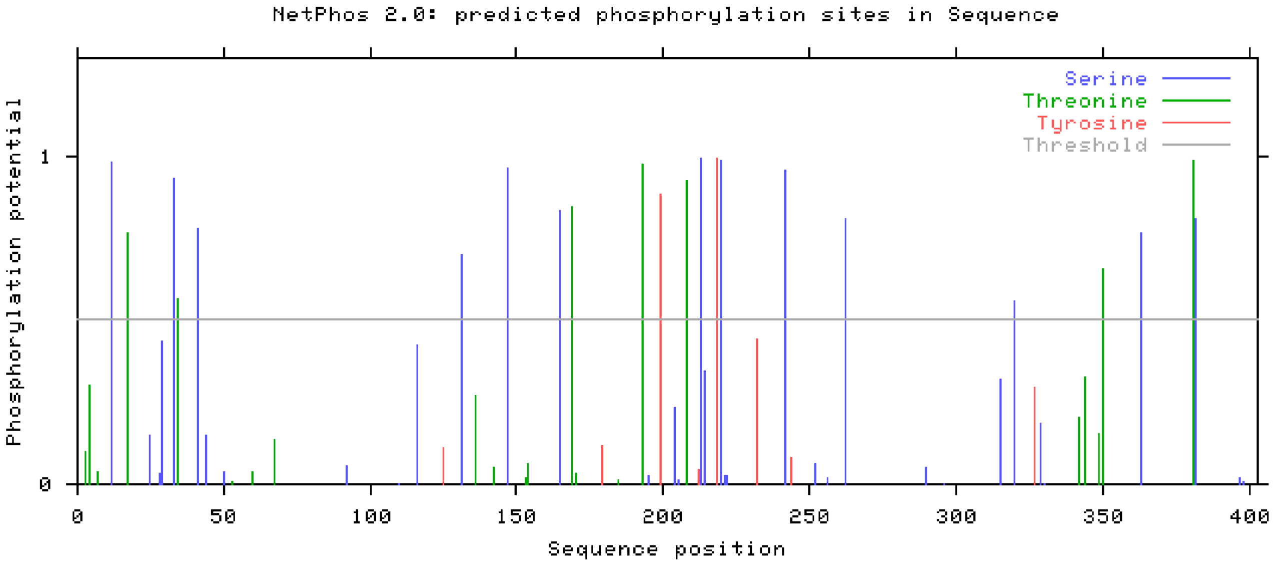

Spot 82, 98 and 141 Beta amylase, partial *T. aestivum* gi|32400764 AAP80614.1

A

```
269 Sequence

GRYPSYPQSHGWSFPGIGEFICYDKYLQADFKAAAAMVGHPEWEFPRDAGQYNDAPQRTRFFVDNGTYLTEQGRFFLAWY     80
SNNLIKHGDKILDEANKVFLGHRVQLAIKISGIHWWYKVPSHAAEITAGYYNLHDRDGYRPIARMLKRHRASLNFTCAEM    160
RDSEQSSQAMSAPEELVQQVLSAGWREGLNMACENALPRYDPTAYNTILRNARPHGINKERAFLSTSWLGFTYLPLXNQX    240
GGGTKTMSISRTFXDRMHANLXYEXMCWS                                                       320
.........................Y.........................Y...............Y............     80
..........................................................Y............S........    160
..S...S...S.....................................................................    240
.......S.S............Y......                                                       320

Phosphorylation sites predicted:     Ser: 6    Thr: 0    Tyr: 5
```

B

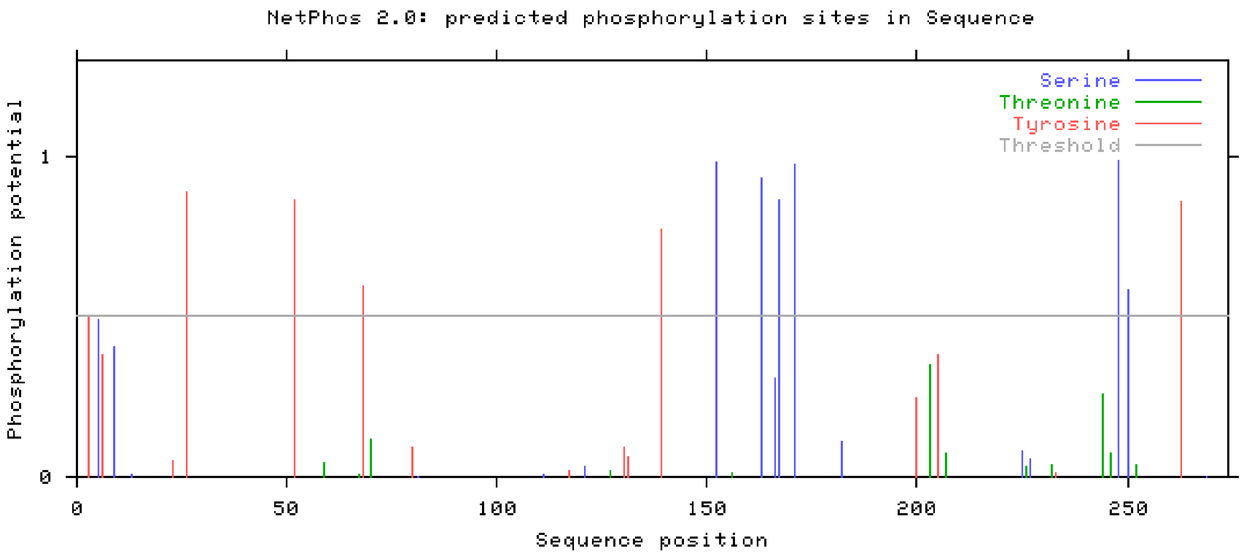

Spot 102 and 301 Beta-amylase *T. aestivum* gi|1771782 CAA67128.1

A

```
503 Sequence
MAGNMLANYVQVYVMLPLDVVSVDNKFEKGDEIRAQLKKLTEAGVDGVMIDVWWGLVEGKGPKAYDWSAYKQVFDLVHEA      80
GLKLQAIMSFHQCGGNVGDVVNIPIPQWVRDVGATDPDIFYTNRGGTRNIEYLTLGVDDQPLFHGRTAVQMYADYMASFR     160
ENMKKFLDAGTIVDIEVGLGPAGEMRYPSYPQSQGWVFPGIGEFICYDKYLEADFKAAAAKAGHPEWELPDDAGEYNDTP     240
EKTQFFKDNGTYLTEKGKFFLSWYSNKLIKHGDKILDEANKVFLGCRVQLAIKISGIHWWYRVPNHAAELTAGYYNLDDR     320
DGYRTIARMLTRHHASMNFTCAEMRDSEQSEEAKSAPEELVQQVLSAGWREGLHVACENALGRYDATAYNTILRNARPKG     400
INKNGPPEHKLFGFTYLRLSNELLEGQNYATFQTFVEKMHANLGHDPSVDPVAPLERSKPEMPIEMILKAAQPKLEPFPF     480
DKNTDLPVKDHTDVGDEVLVAPV                                                              560
................................................................................      80
........................................Y..........Y............................     160
............................S....................Y.........................Y..T.     240
...........Y.T.......S..........................................................     320
..Y............S..........S..S....S.............................................     400
...................S........Y....T.......................S......................     480
.......................                                                              560

Phosphorylation sites predicted:       Ser: 8    Thr: 3    Tyr: 7
```

B

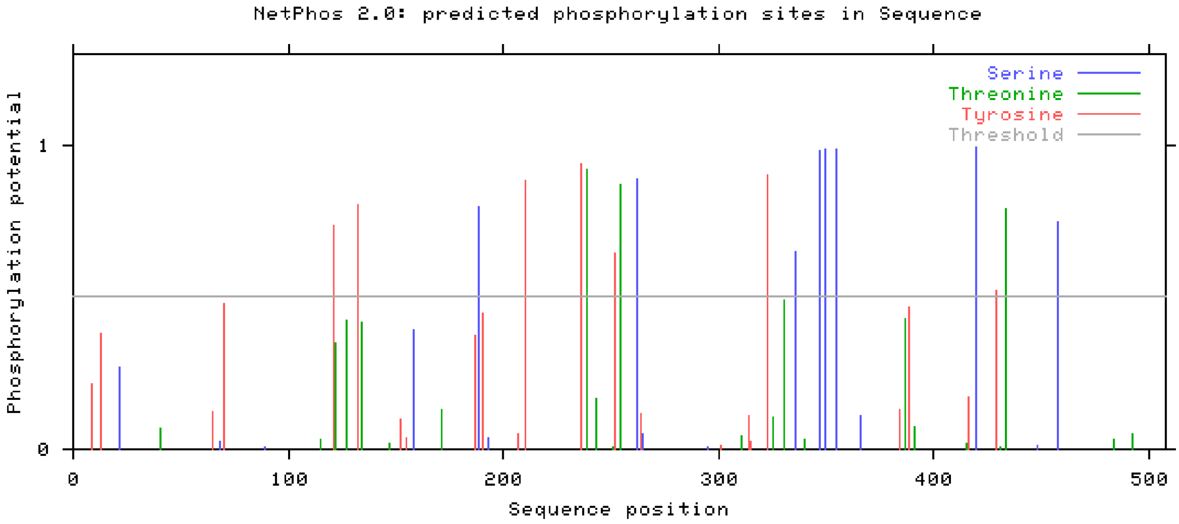

Spot 269 Plastid ADP-glucose pyrophosphorylase small subunit *T. aestivum* gi|224021585 ACN32622.

A

```
514 Sequence
MAMAAAASPSKILIPPHRASAATAAASTSCDSFRLLCAPRGRRQRPRGLVARSAPRRPFFFSPRAVSDSKSSQTCLDPDA      80
STSVLGIILGGGAGTRLYPLTKKRAKPAVPLGANYRLIDIPVSNCLNSNISKIYVLTQFNSASLNRHLSRAYGSNIGGYK     160
NEGFVEVLAAQQSPDNPDWFQGTADAVRQYLWLFEEHNVMEYLILAGDHLYRMDYEKFIQAHRETDADITVAALPMDEER     240
ATAFGLMKIDEEGRIIEFAEKPKGEQLKAMMVDTTILGLDDARAKEMPYIASMGIYVISKHVMLQLLREQFPGANDFGSE     320
VIPGATSTGMRVQAYLYDGYWEDIGTIEAFYNANLGITKKPIPDFSFYDRSAPIYTQPRHLPPSKVLDADVTDSVIGEGC     400
VIKNCKIHHSVVGLRSCISEGAIIEDTLLMGADYYETEADKKLLAEKGGIPIGIGKNSHIKRAIIDKNARIGDNVMIINV     480
DNVQEAARETDGYFIKSGIVTVIKDALLPSGTVI                                                   560
.......S..................ST.................................S....S.S..S........      80
....................T.........................................................Y.     160
............S.........T.........................................................     240
.T.....................................................Y........................     320
......S......................................S........Y..................S......     400
..................................Y.............................................     480
............Y.....................                                                   560

Phosphorylation sites predicted:        Ser: 10    Thr: 4    Tyr: 5
```

B

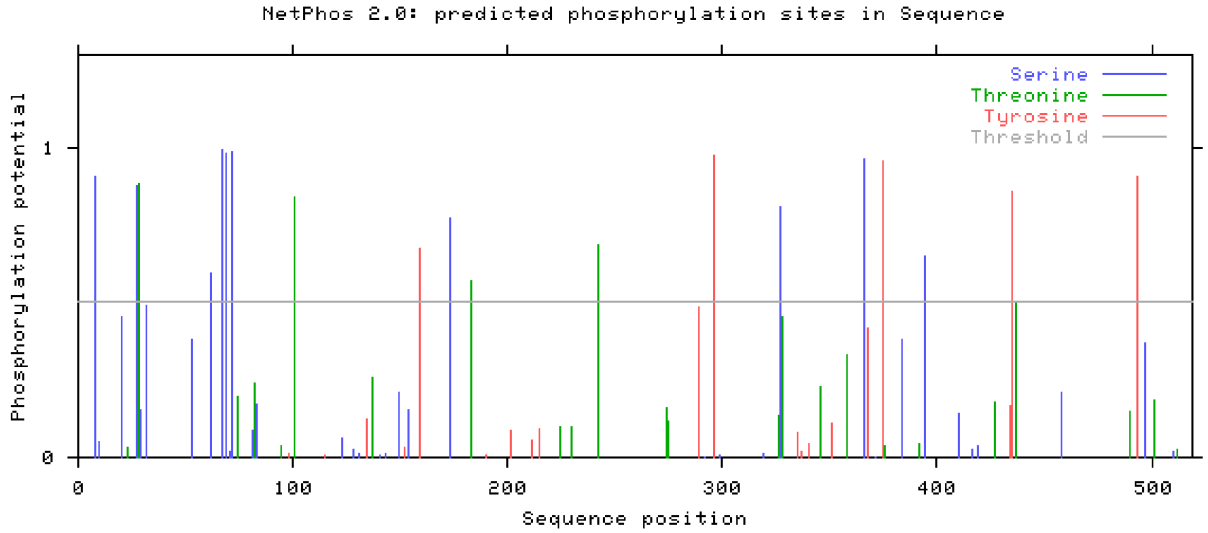

Supplement: Supplementary file 11 [file Image3.PDF]
