# Supplementary material for: Proteomic Analysis Reveals Key Proteins and Phosphoproteins upon Seed Germination of Wheat (Triticum aestivum L.)
Source: Front Plant Sci. 2015 Nov 18;6:1017. doi: 10.3389/fpls.2015.01017 (PMC4649031; doi:10.3389/fpls.2015.01017)

Supplemental Figure S4. Sequence alignment of phosphorylated modification sites in P^{3}DB database on DEPs in germination.

Spot 6 Sucrose synthase type I *T. aestivum* gi|3393067 CAA04543.1

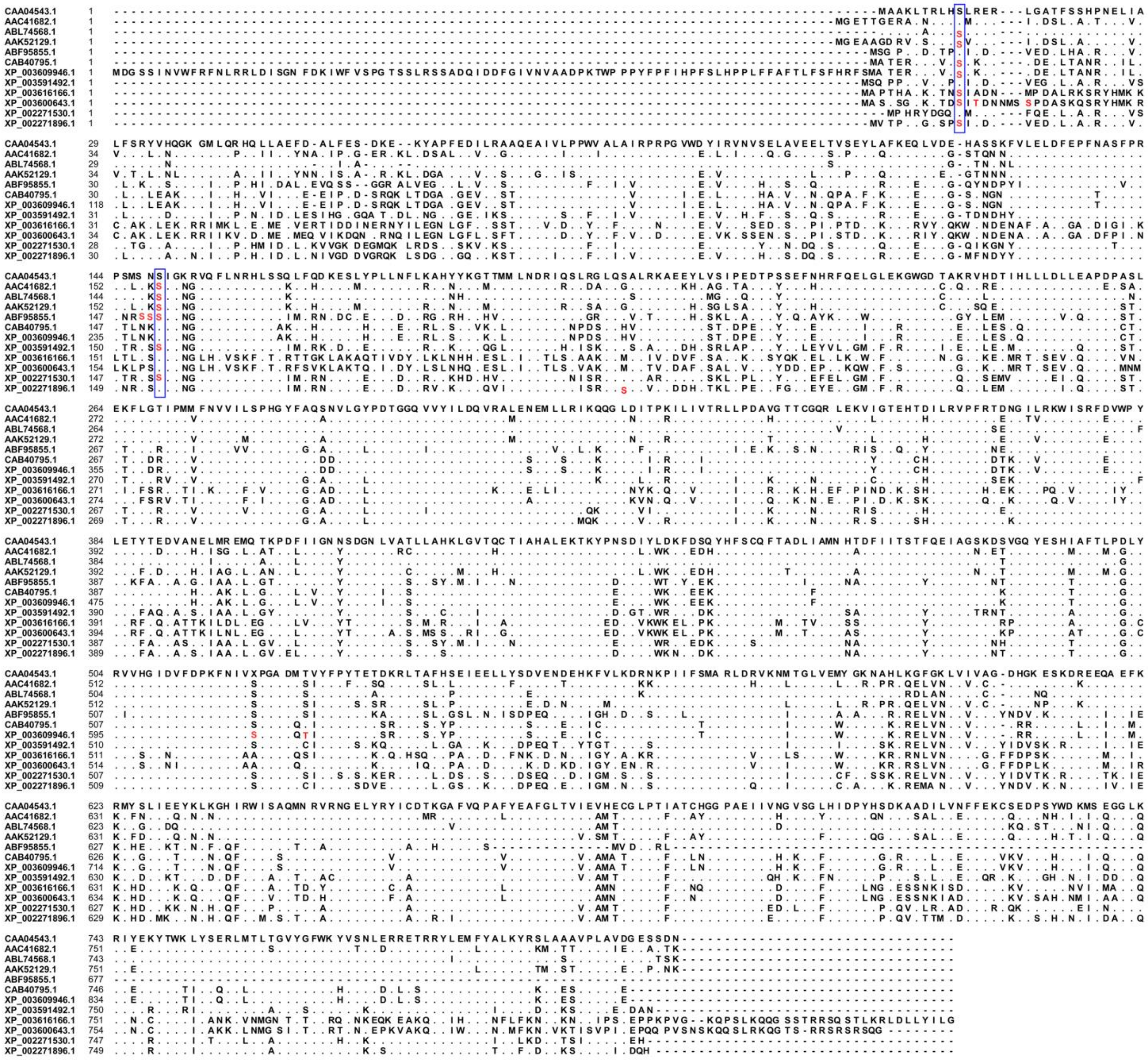

Spot 39 and 232 Serpin *T. aestivum* gi|1885346 CAA72274.1

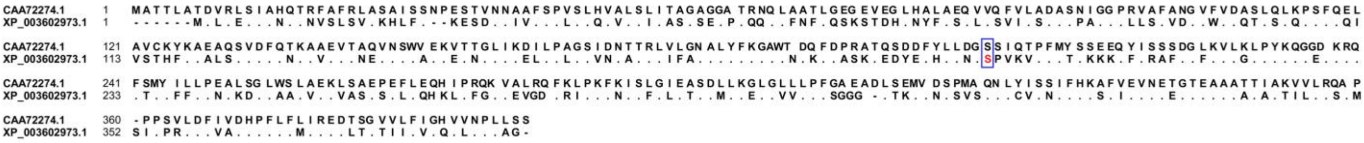

Spot 82, 98 and 141 Beta amylase, partial *T. aestivum* gi|32400764 AAP80614.1

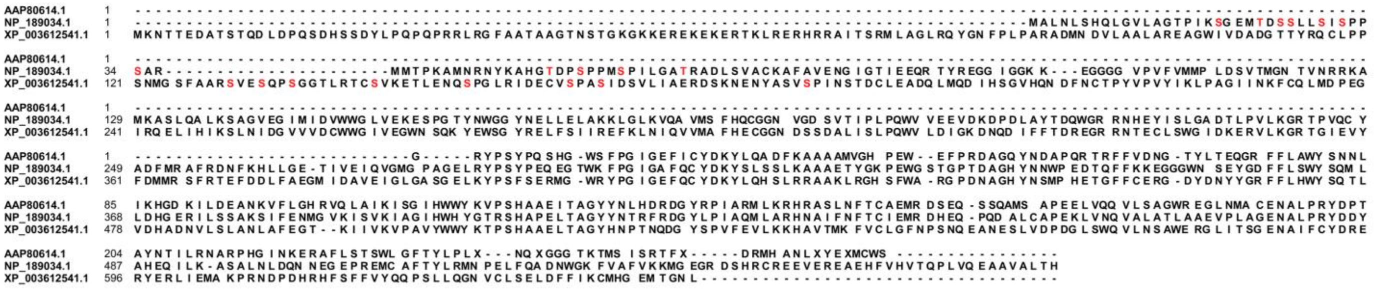

Spot 102 and 301 Beta-amylase *T. aestivum* gi|1771782 CAA67128.1

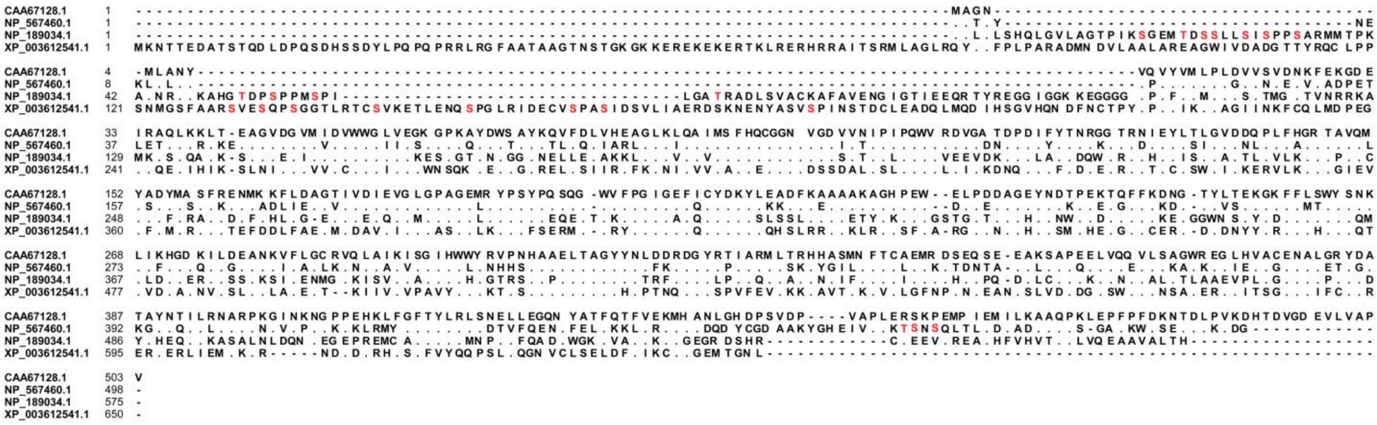

Spot 269 Plastid ADP-glucose pyrophosphorylase small subunit *T. aestivum* gi|224021585 ACN32622.1

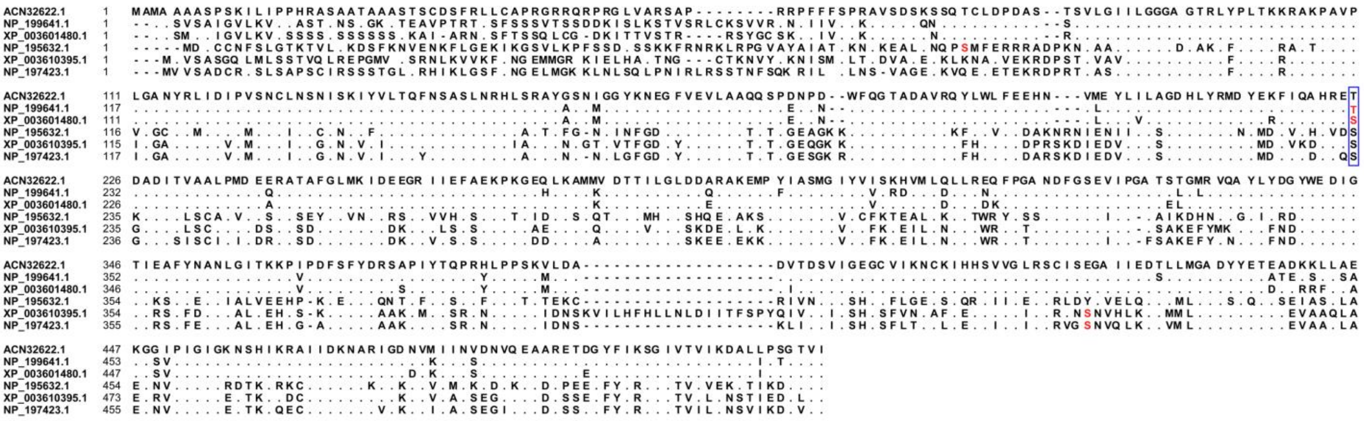

Supplement: Supplementary file 12 [file Image4.PDF]
